# Comparative whole-genome resequencing to uncover selection signatures linked to litter size in Hu Sheep and five other breeds

**DOI:** 10.1186/s12864-024-10396-x

**Published:** 2024-05-15

**Authors:** Tao Zhong, Dunying Hou, Qianjun Zhao, Siyuan Zhan, Linjie Wang, Li Li, Hongping Zhang, Wei Zhao, Shizhong Yang, Lili Niu

**Affiliations:** 1 Farm Animal Genetic Resources Exploration and Innovation Key Laboratory of Sichuan Province, Sichuan Agricultural University, Chengdu, 611130 China; 2 Institute of Animal Science, Chinese Academy of Agricultural Sciences (CAAS), Beijing, 100193 China; 3 College of Animal Science, Xichang University, Xichang, 615013 China; 4 Academy of Agricultural Sciences Liangshan, Xichang, 615000 China

**Keywords:** Hu sheep, Whole-genome resequencing, Selection signatures, Litter size

## Abstract

Hu sheep (HS), a breed of sheep carrying the *FecB* mutation gene, is known for its “year-round estrus and multiple births” and is an ideal model for studying the high fecundity mechanisms of livestock. Through analyzing and comparing the genomic selection features of Hu sheep and other sheep breeds, we identified a series of candidate genes that may play a role in Hu sheep’s high fecundity mechanisms. In this study, we conducted whole-genome resequencing on six breeds and screened key mutations significantly correlated with high reproductive traits in sheep. Notably, the *CC2D1B* gene was selected by the fixation index (*F*_ST_) and the cross-population composite likelihood ratio (XP-CLR) methods in HS and other five breeds. It was worth noting that the *CC2D1B* gene in HS was different from that in other sheep breeds, and seven missense mutations have been identified. Furthermore, the linkage disequilibrium (LD) analysis revealed a strong linkage disequilibrium in this specific gene region. Subsequently, by performing different grouping based on *FecB* genotypes in Hu sheep, genome-wide selective signal analysis screened several genes related to reproduction, such as *BMPR1B* and *PPM1K*. Besides, *F*_ST_ analysis identified functional genes related to reproductive traits, including *RHEB*, *HSPA2*, *PPP1CC*, *HVCN1*, and *CCDC63*. Additionally, a missense mutation was found in the *CCDC63* gene and the haplotype was different between the high reproduction (HR) group and low reproduction (LR) group in HS. In summary, we discovered genetic differentiation among six distinct breeding sheep breeds at the whole genome level. Additionally, we identified a set of genes which were associated with reproductive performance in Hu sheep and visualized how these genes differed in different breeds. These findings laid a theoretical foundation for understanding genetic mechanisms behind high prolific traits in sheep.

**Supplementary Information:**

The online version contains supplementary material available at 10.1186/s12864-024-10396-x.

## Introduction

Sheep (*Ovis aries*) have been a foundational source of meat, wool, and milk for humans, representing one of the most economically significant livestock for thousands of years [1]. The reproductive trait holds significant economic value for sheep production, regulated by heredity [2], hormone [3], environment [4], and managing factors. Previous studies have demonstrated the pivotal role of genetic factors in determining the reproductive performances of sheep. Elucidating the genetic mechanisms underlying high prolificacy is crucial for improving sheep production through the utilization of prolific resources. The litter size in sheep was influenced by a combination of major genes and polygenes. Currently, multiple major genes affecting prolificacy have been reported in sheep [5]. Previous studies have demonstrated a positive correlation between litter size and the genotypes of *FecB* [6]. The distribution of *FecB* genotypes was various among breeds and strains [7, 8]. In the high-fertility Hu sheep, some ewes harboring the *FecB* homozygous mutation (c.746 A > G) that delivered a single lamb, indicating additional genetic factors resulted in a reduction in litter sizes. Screening for fertility genes is helpful for marker-assisted breeding to increase the performance of reproduction in sheep. Uncovering the genetic mechanism of sheep prolificacy is conducive to enhancing the reproduction of Chinese sheep breeds.

The genetic mechanisms of morphological and agronomic traits in sheep have been gradually revealed and continue to be studied with the completion of the sheep reference genome [9]. Studies have focused on various aspects, including the domestication and chromosome evolution in wild sheep [10]. Additionally, researchers have delved into important traits, such as reproduction, coat color [11], horn phenotype [12], tail fat deposition [13], and body size [14]. Whole-genome resequencing could identify the selective traits behind phenotypic differences and reveal the genetic basis of complex traits [13]. Identifying the genetic variations responding to different phenotypes could clarify the genetic mechanisms underlying high productivity in varieties. The acquisition of genome-wide genetic mutations provided a solid foundation for establishing genomic selective breeding [15]. The primary work is to further explore reproduction-related genes and functional loci to supply favorable conditions for exploring the genetic underpinnings of high fecundity and new variety breeding. While the main relevant major genes affecting the reproductive traits have been identified, such as *BMP15* [16, 17], *GDF9* [17, 18], *B4GALNT2* [5], and *KISS1R* [19], their genetic roles in most sheep breeds remained unclear. In addition, the candidate genes affecting litter size varied among breeds and resulted from the combined effects of multiple candidate genes. Thus, further investigation is necessary to uncover the key genes regulating reproductive traits in sheep [20–22]. Hu sheep, a prolific native breed in China, serve as valuable animal models for understanding the genetic underpinnings of prolific sheep breeds [23]. Furthermore, analyzing the genome data in Hu sheep could provide new insight into differences in reproduction performance, potentially enabling the replication of the multiple birth characteristics of Hu sheep in other sheep breeds [24].

Lacking evidence to clarify the regulatory mechanisms and candidate genes related to reproductive traits, we performed the whole-genome resequencing of Hu sheep and Liangshan Black sheep (LB), and jointly analyzed with the genome-wide data of Bamei Mutton sheep (BM), White Xizang sheep (WX), Oula sheep (OL), and Poll Dorset sheep (PD) aimed to explore the molecular basis of the high fertility trait, as well as conducting a comparative analysis of genome-wide DNA SNPs among individuals with different *FecB* genotypes. We identified genomic regions and key mutations significantly associated with high fertility traits in Hu sheep and validated in various breeds with different fertility levels. In addition, our findings contribute to the exploration of high-quality genes related to high fertility in Hu sheep and provide insight into the molecular regulatory mechanisms underlying reproduction among different sheep breeds.

## Materials and methods

### Sampling collection and DNA extraction

274 ewes of Hu sheep were collected from the sheep farm of the Yuexi Oriental Agricultural Development Co., LTD (Liangshan, Sichuan, China). 53 female Liangshan Black sheep were randomly selected from the Butuo County Black Sheep Breeding Farm (Liangshan, Sichuan, China). Whole blood was collected from the jugular vein and kept in EDTA vacutainer tubes. The genomic DNA of blood sample was extracted using a Genomic DNA Kit (TansGen Biotech, Beijing, China). The integrity and concentration of DNA samples were assessed using 1% agarose gel electrophoresis and a NanoDrop 2000 spectrophotometer (Thermo Fisher Scientific, Waltham, MA, USA). Extracted DNA samples were stored at -20 °C prior to further analysis.

### Genotyping of the c.746 A > G locus of *FecB* gene

A fragment of 328 bp harbored the c.746 A > G locus was amplified by the primer pair (Forward 5′-CAGATGGTGAAACAGATTG-3′, Reverse 5′-CAAGTCCACCATCCATTC-3′), which was designed by Primer Premier 5.0 software and synthesized by Sangon Biotech Co., Ltd (Shanghai, China). Polymerase chain reaction (PCR) was conducted on a BIO-RAD C1000Touch^™^ Thermal Cycler (CA, USA) under the following conditions: 95 °C for 3 min and 34 cycles of 30 s at 95 °C, 30 s at 55 °C, 23 s at 72 °C, and a final step of 5 min at 72 °C, then stored at 4 °C. The PCR products were purified and sequenced by an ABI 3730 Sequencer (Thermo Fisher Scientific, Waltham, MA, USA) to determine the *FecB* genotype (c.746 A > G) in all HS and LB individuals. In addition, the genotype of the c.746 A > G locus in the other joined analyzed breeds (BM, WX, OL, and PD) were determined by the whole genome resequencing data.

### Whole genome resequencing and data processing

29 HS and 19 LB were randomly selected for whole genome resequencing. The paired-end sequencing libraries with an insert size of ∼ 350 bp fragments for each individual were constructed to ∼10× raw coverage using the BGISEQ DNBSEQ-T7 platform (BGI lnc., Shenzhen, China) according to the manufacturer’s protocol at Novogene Bioinformatics Technology Co., Ltd (Beijing, China). In addition, resequencing data of BM, WX, OL, and PD were obtained from the previous studies [25, 26].

All fastq files were processed through the use of fastp (v0.19.5) to obtain clean reads. The following filter criteria were employed to eliminate adapters and low-quality bases: reads with more than 10% unknown nucleotides (N), reads with over 50% low-quality bases (Q-value < 5), and reads with more than 10 nucleotides aligned to the adaptor sequence with a maximum of two mismatches. The resulting clean reads were then mapped to the sheep Oar_v4.0 reference genome utilizing Burrows-Wheeler Aligner v0.7.17 (BWA) algorithm with default parameters [27]. Mapping files were converted to BAM files and sorted using SAMtools (v1.9). Single nucleotide polymorphism (SNP) calling was performed using the Bayesian method in the GATK package (v4.1.3.0–0) with the subsequent filtering criteria: QD < 10.0, ReadPos RankSum < -8.0, FS > 10, QUAL < 30, and DP < 4. VCFtools (v0.1.14) was utilized to generate a raw VCF file, which was then filtered to create a high-quality VCF file with retained SNPs for further analysis. The filtering criteria for the high-quality VCF file were as follows: (1) %QUAL < 100 and (2) INFO/DP < 5. To annotate the SNPs and indels, ANNOVAR was employed, utilizing gene models from GFF annotation [28].

### Genetic diversity and runs of homozygosity (ROH)

Nucleotide diversity (*π*) for each breed was investigated using VCFtools v.0.1.16 with a 40 kb non-overlapping window across all autosomes [29]. PLINK (--hardy) was used to estimate observed heterozygosity (*Ho*) and expected heterozygosity (*He*). *He* and *Ho* estimates for individuals in each breed were averaged across all SNPs. Long homozygous fragments were scanned by using PLINK according to the following parameters: --homozyg-window-snp 50 --homozyg-snp 50 --homozyg-kb 300 --homozyg-density 50 --homozyg-gap 1000 --homozyg-window-missing 5 --homozyg-window-threshold 0.05 --homozyg-window-het 1 [30]. The genomic inbreeding coefficient based on ROH (*F*_ROH_) is the ratio of the total length of ROH fragments in the genome to the total length of the permanent genome, and each ROH is classified according to its physical length as follows: 0–1, 1–4, and ≥ 4 Mb.

### Population structure analysis

Population genomics analyses was used to explore the genetic relationship of individuals from HS, LB, BM, WX, OL, and PD, SNPs that did not meet one of the following criteria (--maf 0.05 --max-missing 0.9) were filtered by PLINK v1.9 [31]. Additionally, the linkage disequilibrium (LD) of each sheep population was removed with the criteria: --indep-pairwise 50 10 0.1. Principal components analysis (PCA) was applied to visualize patterns in relationships between six sheep breeds. Furthermore, population structure was examined using ADMIXTURE v.1.23 to estimate the cross-error for genetic clustering with the ancestral clusters (*K*) ranging from 2 to 5 and each *K* was run the analysis for 100 times [32]. The SNPs were used to calculate distance matrix by the software VCF2Dis to construct the phylogenetic tree. The topological structure was displayed by the Interactive Tree of Life (ITOL) tool (https://itol.embl.de/) [33]. The linkage disequilibrium (LD) r^2^ with physical distance between SNPs for each breed was calculated using PopLDdecay with default parameters [34].

### Selective signatures related to litter size

We identified genome-wide selective sweeps during all breeds based on *F*_ST_ analysis using VCFtools. The *F*_ST_ values were calculated using a sliding window approach, with 80 kb windows and 40 kb sliding steps according to the previous study [35]. Windows with *F*_ST_ values which were > 0.25 were defined as the putatively selected genomic regions. For the fecundity analysis of Hu sheep, we combined the LB, OL, WX, BM, and PD into a group (others) and compared them with HS using two different statistics, including *F*_ST_ and XP-CLR with size 80 kb windows and 40 kb step. Overlapping regions that *F*_ST_ > 0.25 [36] and XP-CLR score > 2.5 were identified to be candidate regions. The selective sweeps of Hu sheep breeds were also detected by comparison with different litter size. The haplotype blocks based on vcf file were analyzed by LDBlockShow [37]. Moreover, the KOBAS 3.0 (http://kobas.cbi.pku.edu.cn/) was utilized to conduct the Kyoto Encyclopaedia of Genes and Genomes (KEGG) analysis.

## Results

### Whole genome resequencing, SNP calling, and genetic diversity

We conducted whole-genome resequencing on a total of 86 sheep samples (Table S1). The sequencing data had an average coverage depth of ∼ 10.31× for HS and LB, ∼ 5.76× for the previously characterized 39 individual genomes. After aligning the clean reads to the reference genome, we identified a total of 39,467,233 putative SNPs and 8,677,193 indels in the six breeds, 50.1% of which were located within introns and 41.1% within intergenic regions. A total of 26,664,234 SNPs were retained after quality filtering.

To gain insights into the genetic diversity of the six sheep breeds, *He*, *Ho*, and the numbers of ROH were estimated based on genotype frequencies (Table 1). The highest *He* value was observed in HS, indicating a greater level of genetic diversity within the population, while the lowest values were found in WX. The *Ho* within the population ranged from 0.1828 to 0.2395, respectively, with the lowest values in BM and the highest in LB. Apart from that, ROH analysis revealed that HS and LB had 14 and 38 long ROH segments, respectively. Meanwhile, shorter ROH segments exited in OL, WX, BM, and PD. In addition, the LD decay pattern and ROH distribution of each group were roughly consistent with the results of nucleotide diversity. (Fig. S1). The distribution of SNPs in each chromosome was visualized in Fig. S2, presenting a non-uniform distribution with wide coverage.

**Table 1:** Genetic diversity parameters and the runs of homozygosity (ROH) of the six sheep breeds

Breeds	Abbreviation	ROH number MEAN ± SD	ROH number (0–1 MB)	ROH number (1–4 MB)	ROH number (≥ 4 MB)	*F*_ROH_ MEAN ± SD	ALL SNPs	
							*He*	*Ho*
Hu Sheep	HS	268.46 ± 54.41	6904	569	14	0.0528 ± 0.0199	0.3233	0.2282
Liangshan Black Sheep	LB	510.46 ± 79.46	8842	784	38	0.1049 ± 0.0393	0.2835	0.2395
Oula Sheep	OL	238.22 ± 52.38	2066	69	0	0.0389 ± 0.0100	0.2283	0.2205
White Xizang Sheep	WX	190.60 ± 62.62	1834	63	0	0.0310 ± 0.0117	0.2175	0.2113
Bamei Mutton Sheep	BM	235.50 ± 112.25	2152	197	0	0.0458 ± 0.0249	0.2266	0.1828
Poll Dorset Sheep	PD	202.20 ± 89.26	1855	163	1	0.0393 ± 0.0210	0.2248	0.1959

### Population-level analyses of genetic structure and LD decay

**Fig. 1:**
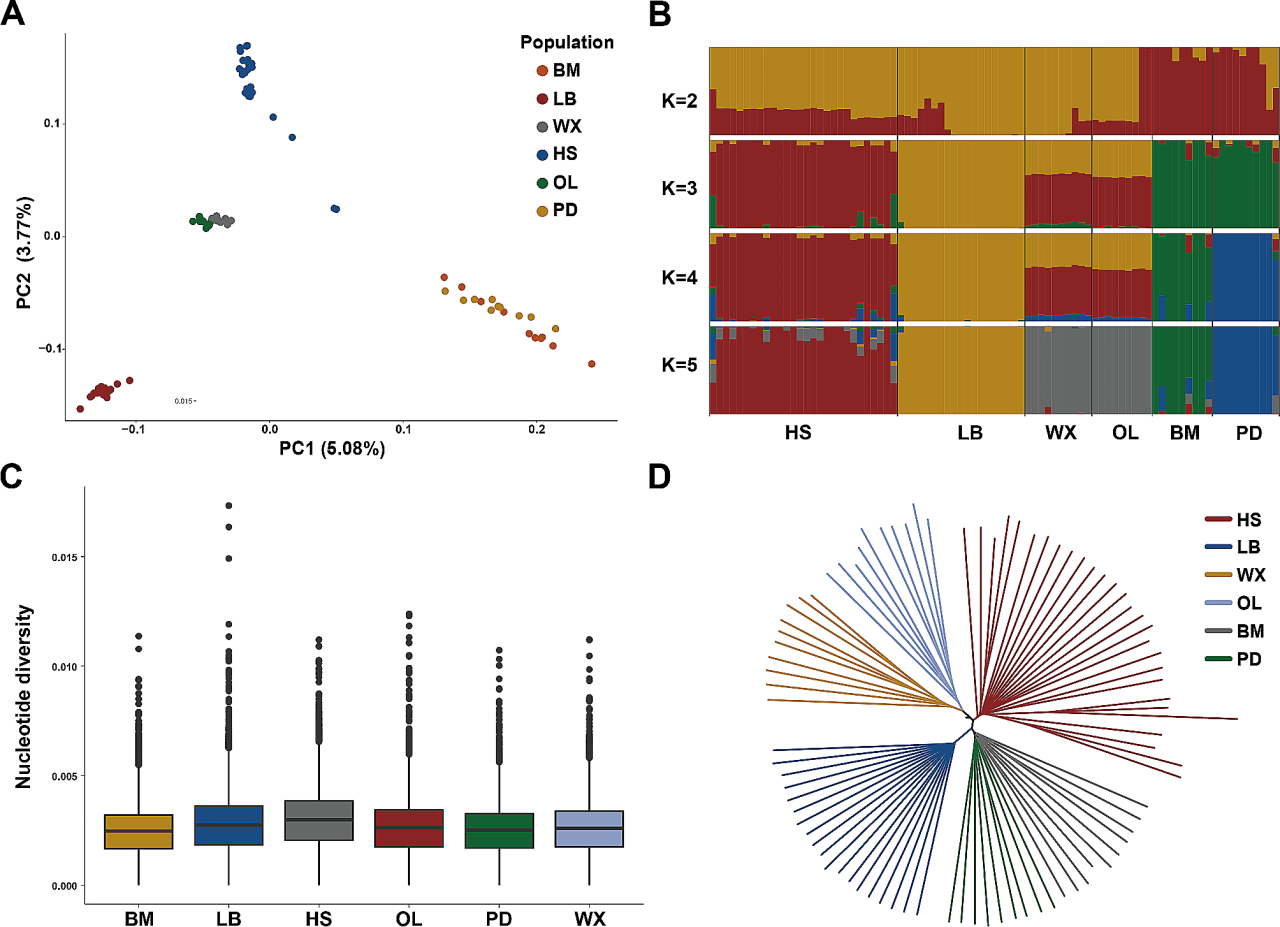
Population genetic analysis of 6 breeds. (**A**) Principal component analysis of 86 individuals. (**B**) Population genetic structure of sheep. (**C**) Genome-wide distribution of nucleotide diversity of each breed. The points which were on the outside the of whiskers showed outliers. (**D**) Neighbor-joining (NJ) trees of sheep based on whole-genome SNPs

Principal component analysis showed that all sheep clustered into four distinct genetic groups that were completely separate from each other, while WX and OL that belonged to Tibetan sheep were clustered together (Fig. 1A). BM and PD were inextricably interwoven as a branch. Population structure analysis, aiming to estimate the proportion of common ancestry among the six breeds, yielded five genetic clusters at the optimal number *K* = 5 (Fig. 1B). We observed that WX and OL were in the same genetic clusters, BM and PD population tended towards completely distinct layers while *K* = 4 and *K* = 5, these results were consistent with PCA. To further verify the relationships among breeds, NJ tree results showed that all individuals of the same species formed their own clusters (Fig. 1D). Obviously, HS were separated in a single cluster distinctively, meanwhile, OL and WX were in a branch which formed a main branch with LB. On the other hand, BM and PD were in the same branch, which were in agreement about the result of PCA. LD decay analysis suggested that HS showed minimum LD values at larger physical distances, followed by LB and WX (Fig. S1). The LD decay in OL, PD, and BM had the maximum values. In addition, we observed a relatively low level of nucleotide diversity in BM (0.002448429), the average value of nucleotide diversity in HS (0.002958546) was higher than that in other groups (Fig. 1C).

### Selection signals and candidate genes related to prolificacy

**Fig. 2:**
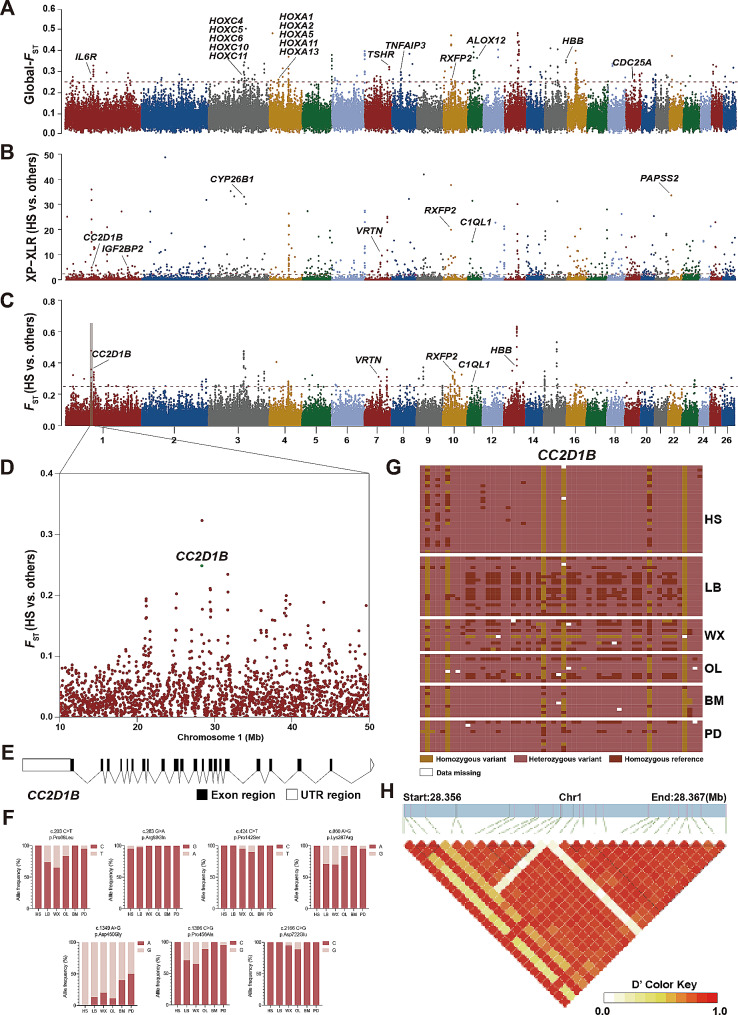
Manhattan plot of selective sweeps by *F*_ST_ and XP-CLR methods in Hu sheep vs. others. (**A**) Genome-wide distribution of *F*_ST_, which is measured by the average value for each SNP across 5 breeds. (**B**) XP-CLR scores from sheep to two groups: HS, other sheep breeds (LB, WX, OL, BM, PD). (**C**) Fixation index (*F*_ST_) values from sheep to two groups: HS, other sheep breeds (LB, WX, OL, BM, PD). (**D**) Plot of *F*_ST_ windows of chr1 region (10–50 Mb) based on HS and others. (**E**) Structures of *CC2D1B* gene. (**F**) Seven missense mutations of *CC2D1B* gene. (**G**) Haplotype differentiation patterns of *CC2D1B* gene. (**H**) Linkage disequilibrium analysis of SNPs in *CC2D1B* region

Figure 2A presented the positive genome selections for *F*_ST_ in six breeds. Thresholds set at *F*_ST_ > 0.25 uncovered substantial sites with 71 putative selective sweeps accounting for 211 genes exhibiting positive selection within six breeds, as detailed in Table S2. Furthermore, KEGG Pathway analysis identified the specific pathways in which selected genes were involved (Table S3). We detected candidate genes related to reproduction (e.g., *TSHR*, *THRA*, *CDC25A, RARA*), what’s more, *MED24* and *THRA* played a role in thyroid hormone signaling pathway which was closely related to reproduction. *RARA* was involved in the estrogen signaling pathway. *CDC25A* was found to be enriched in progesterone-mediated oocyte maturation. *RIPK1* and *TNFAIP3* were found associated with NF-kappa B signaling pathway. Genes associated with disease and immunity, including *GMDS*, *TNFAIP3, LRRK2*, and *DDB1*, were identified in several candidate genomic regions under selection. Selective sweep analysis also captured candidate genes associated with adaptation to the plateau, of these, *IL6R* was involved in HIF-1 signaling pathway. Meanwhile, positive selection was detected in *HoxA* and *HoxC* gene cluster. In addition, we also identified the candidate genes related to other traits, such as the high-altitude hypoxic adaptation (*HBB*) and regulation of horn growth and development (*RXFP2*).

### Shared selected and candidate genes in Hu sheep

For screening out the associated genetic variation, we estimated the *F*_ST_ values and XP-CLR scores to investigate specific genomic regions associated with HS, in comparison to a collective group of five other breeds: LB, WX, OL, PD, and BM. The windows selected as potentially positively selected regions were those simultaneously with both *F*_ST_ values > 0.25 and XP-CLR scores > 2.5. A total of 17 regions, encompassing 39 genes, were found to have undergone positive selection in the populations (Fig. 2B, C, Table S4). Genes associated with reproduction (*CCDC103, CC2D1B, C1QL1*), cell growth *(LIN52, CRADD*), growth and development (*VRTN, C1QL1*), immune function (*KLRF1, MAP3K6*), and other traits were identified. *PAPSS2* was found to be enriched in pathways associated with metabolism, including sulfur metabolism, selenocompound metabolism, and purine metabolism. What’s more, *LIN52* was involved in cellular senescence (Table S5).

*CC2D1B* was observed to have a high *F*_ST_ value and XP-CLR scores on chromosome 1 (Fig. 2D), we performed genotype imputation and visualized the data for *CC2D1B*, revealing variations in this gene among different sheep breeds. Meanwhile, the genetic composition of BM and PD was similar, while the genetic composition of HS was distinct from the other five breeds. Additionally, we identified seven missense mutations in *CC2D1B* and found that the allele frequency of each locus differed among breeds (Fig. 2E, F). Among them, the frequency of c.1349 A > G in HS reached 100%, while up to 86.84% and 88.89% in LB and OL, respectively. Visualizing the *CC2D1B* genotype revealed variations among different breeds (Fig. 2G), and haplotype analysis indicated a significant imbalance in linkage within this region (Fig. 2H).

### Selection prints in Hu sheep harbored different *FecB* genotypes

To compare the distribution of *FecB* among different breeds, we examined the *FecB* genotypes of 366 sheep (Table S6). The results showed that there were three genotypes BB (79.9%), B+ (18.6%) and ++ (1.5%) in HS, and only two genotypes B+ (20%) and ++ (80%) were found in WX, and only + + genotype was detected in LB, OL, BM, and PD.

**Fig. 3:**
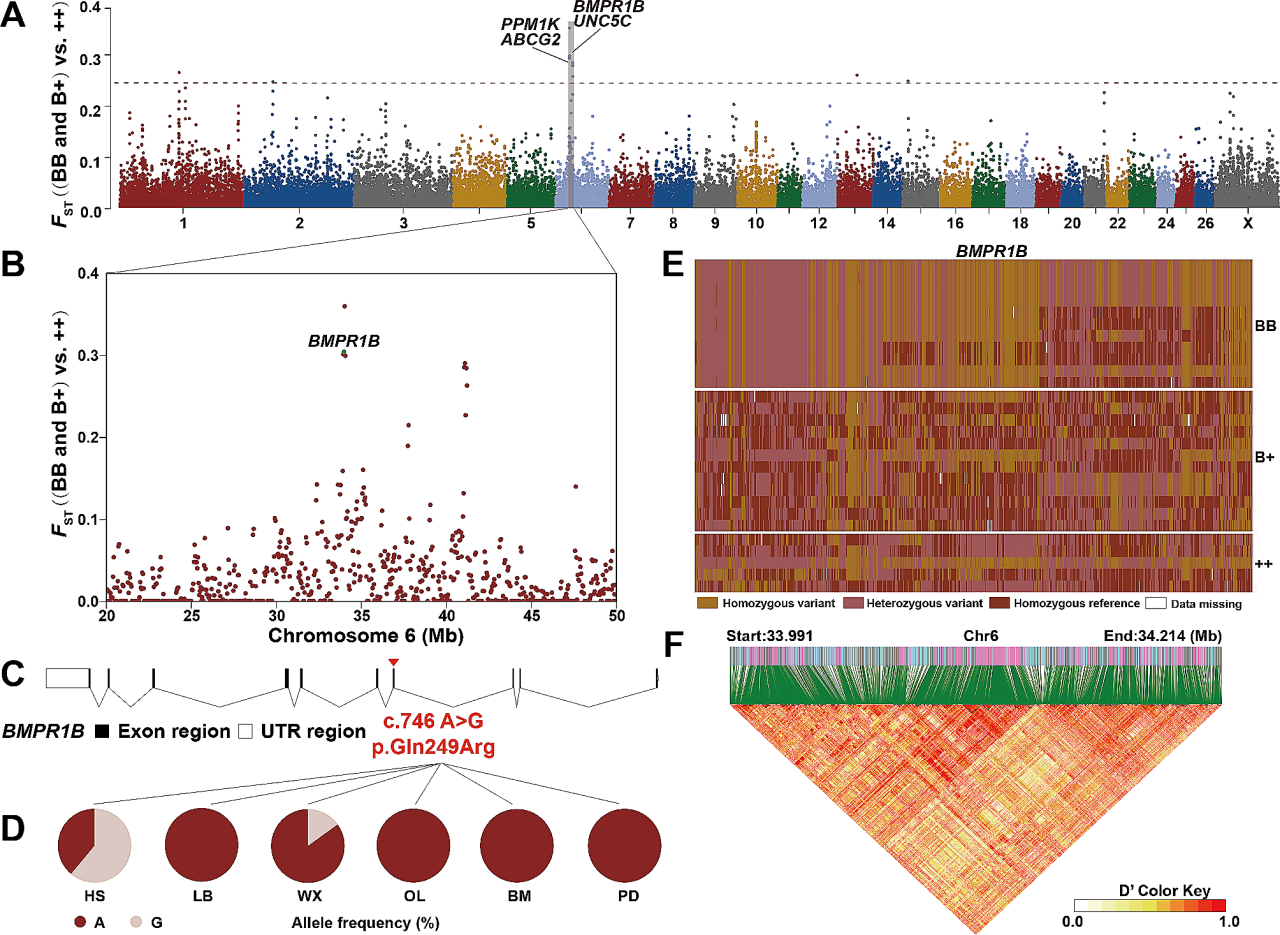
Genome-wide annotations during sheep of reproduction. (**A**) Manhattan plot of genome-wide selection signatures associated with *FecB* genotypes in Hu sheep. (**B**) Plot of *F*_ST_ windows of chr6 region (20–50 Mb) based on different *BMPR1B* genotypes in Hu sheep. (**C**) Structures of *BMPR1B* gene. (**D**) Allele frequencies of *BMPR1B* gene c.746 A > G. Locations of the SNPs were determined by the generated VCF file. (**E**) Haplotype diversity of a local region of *BMPR1B* (chromosome 6: 33,990,928–34,214,488). (**F**) Linkage disequilibrium analysis of SNPs in *BMPR1B* region

*F*_ST_ values were calculated based on different *FecB* genotypes ((BB and B+) vs. ++) to explore critical molecular signals related to the high prolificacy of Hu sheep (Fig. 3A, B). We selected the windows with *F*_ST_ > 0.25 across the genome and identified three unique autosomal regions with the strongest selective signals containing five candidate genes which included *UNC5C*, *U6*, *ABCG2*, *BMPR1B*, and *PPM1K*. The *BMPR1B* (*F*_ST_ = 0.300978) and *PPM1K* (*F*_ST_ = 0.283851), associated with reproduction traits, were identified on chromosome 6: 33,975,001–34,125,000 bp and 41,100,001–41,250,000 bp, respectively. A significant differentiation was observed in genotype profiles of *BMPR1B* among the three groups with different *FecB* genotypes (Fig. 3E). Many strong linkages were detected in *BMPR1B* (Fig. 3F). Within the *BMPR1B* gene, there was a missense mutation c.746 A > G was identified, which resulted in the substitution of Gln with Arg (Fig. 3C). This specific mutation showed the highest occurrence rate in HS at 61.11%, followed by 15% in WX (Fig. 3D). Furthermore, this mutation was not observed in LB, OL, BM, and PD (Fig. 3D).

### Insight into the novel target genes response to high prolificacy in Hu sheep

**Fig. 4:**
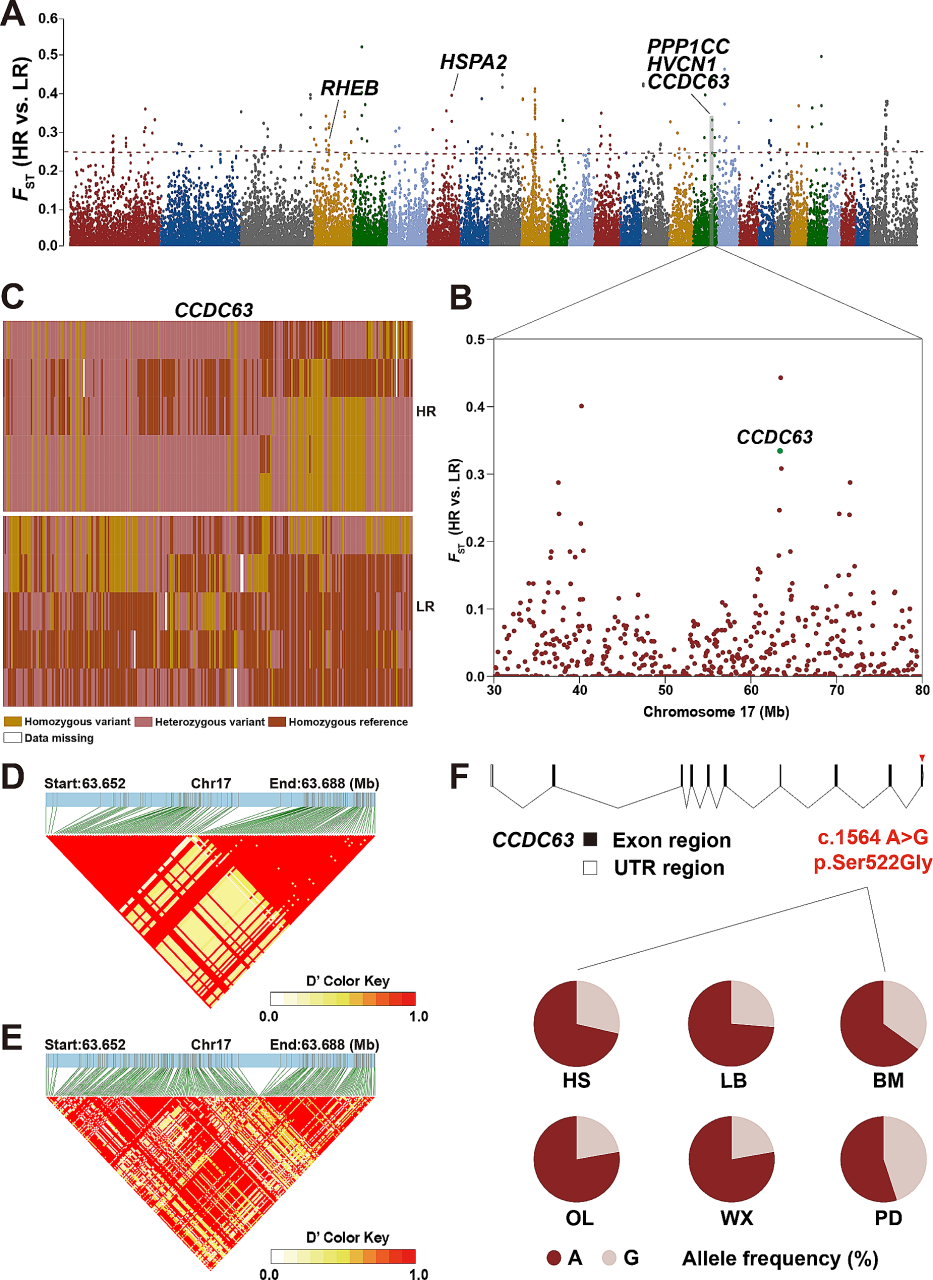
Selection imprints of different litter sizes. (**A**) Identification of candidate genes related to litter size of Hu sheep. (**B**) Plot of *F*_ST_ windows of chr17 region (30–80 Mb) based on HR vs. LR in Hu sheep. (**C**) Haplotype diversity of a local region of *CCDC63* (chromosome 17: 63,652,194–63,687,626) in HR and LR. (**D**) Linkage disequilibrium analysis of SNPs in *CCDC63* gene region of HR group. (**E**) Linkage disequilibrium analysis of SNPs in *CCDC63* gene region of LR group. (**F**) Structures of *CCDC63* gene and allele frequencies of *CCDC63* gene

Previous studies have confirmed that mutations in the *FecB* gene were completely associated with high reproduction. However, we found some HS ewes carrying the homozygous *FecB* alleles still produced single-offspring. To further determine the significance of genomic divergence, we analyzed and compared the genomes of single-lamb and multi-lamb groups of HS (Table 2), representing high reproduction and low reproduction groups, to identify potential selection imprints. 118 genes were identified as under positive selection with *F*_ST_ > 0.25 (Fig. 4A). Particularly, *PPP1CC*, *CCDC63*, *HSPA2*, and *RHEB* which were associated with reproductive traits, indicating functional importance. In addition, genes related to immune diseases (e.g., *BID, SMARCD3, ATP6V1E1, NFKB2*), energy metabolism (e.g., *HAO1, NDUFA7, CERS4, MTHFD1*), and other factors were also screened (Fig. 4B, Table S7). In KEGG analysis, it was found that *PPP1CC* was involved in two reproductive-related biological processes: the oxytocin signaling pathway and oocyte meiosis. *HSPA2* and *RHEB* were identified to participate in the estrogen signaling pathway and thyroid hormone signaling pathway. These pathways played a crucial role in the secretion and regulation of hormones. The KEGG enrichment analysis revealed that candidate genes were significantly enriched in important economic traits, such as reproductive traits, growth and development traits (Fig. 4B, Table S7). Especially, a missense mutation on *CCDC63*, (c.1564 A > G) was identified in all six breeds (Fig. 4D), what’s more, haplotype diversity and linkage disequilibrium analysis were performed to detect mutations in gene regions (Fig. 4C, D, E). Simultaneously, allelic frequencies of the c.1564 A > G locus in *CCDC63* were calculated within the 86 individuals of the six breeds (Fig. 4F). The allele frequency in PD was the highest at 45%, while the allele frequency in WX and OL was the lowest at 28.57% (Fig. 4F). KEGG enrichment analysis detected top 30 enriched signaling pathways relevant to candidate genes. Some known pathways related to immunity were found to be significantly enriched (Fig. S3, Table S8).

**Table 2:** The classification information of different reproduction of Hu sheep

High Reproduction Group (HR)			Low Reproduction Group (LR)		
Individual ID	Litter size	*FecB* Genotype	Individual ID	Litter size	*FecB* Genotype
H3	5	BB	H7	1	B+
H6	5	BB	H8	1	B+
H12	4	BB	H9	1	B+
H13	4	BB	H10	1	B+
H15	4	BB	H16	1	BB

## Discussion

Hu sheep is one of the high reproductive breeds in China, the occurrence of multiple births in Hu sheep is still not clearly understood. Some scholars speculated that it may be influenced by human selection and historical migration patterns [38]. Others suggested that the occurrence of multiple births in Hu sheep was determined by specific genotypes that can be inherited by subsequent generations [39].

The characteristics of population genetic diversity were essential for assessing the genetic potential of breeds as well as for the utilization and protection of sheep breed resources. The ranking of nucleotide diversity (*π*) within different breeds was: HS > LB > OL > WX > PD > BM. One possible explanation for the high nucleotide diversity in HS could be that Hu sheep, with a larger effective population size, originated from the northern Mongolian sheep. Through years of domestication and breeding in a favorable environment, they acquired genetic information from multiple sources [40]. Beyond that, OL and WX exhibited a close genetic relationship, likely due to their proximity geographically and intentional breeding practices [41]. The WX breed was originally bred by Tibetans specifically in the high-altitude environment of the Tibetan Plateau. Known for its superior wool quality, the breed exhibited relatively lower meat productivity. OL, on the other hand, originated from local Tibetan sheep and wild sheep [42, 43]. Genetic diversity, as an essential foundation of biodiversity, was the result of long-term species survival, evolution, and adaptation. This study found that the domestic sheep breed, HS, LB, OL, and WX, exhibited higher genetic diversity, while BM and PD with foreign genetic backgrounds, demonstrated lower genetic diversity. These findings were consistent with the previous studies [44]. Additionally, the ROH analysis indicated that HS and LB, which exhibit longer ROH segments, have undergone adaptive evolution in response to specific environmental pressures, leading to the selection and fixation of certain alleles that confer an advantage [45]. In contrast, shorter ROH segments in OL, WX, BM, and PD may be related to their population history and lower levels of inbreeding [46]. A lower *F*_ROH_ value suggests a lower degree of inbreeding and potentially a more diverse gene pool [47]. The high *F*_ROH_ and low *He* in LB may suggest a need for strategies to increase genetic diversity and reduce inbreeding to avoid potential issues such as inbreeding depression [46]. On the other hand, the low *F*_ROH_ and high *He* in WX may be beneficial for maintaining a stable population with a rich genetic background [45].

The molecular mechanisms underlying the high prolificacy trait in Hu sheep have not been extensively studied, and so far, no other major molecular markers have been identified except for *FecB*. Genome selection signals have been used to identify genomic regions and genes associated with the reproductive traits of Hu sheep. In our study, a set of genes has been identified through global *F*_ST_ analysis in six breeds (Fig. 2A, Table S2). As previously reported, one of the functions of *TSHR* was to regulate the development of the thyroid gland and the secretion of thyroid hormone, which in turn plays an important role in the seasonal reproduction of mammals [48, 49]. *TSHR* could catalyze cAMP synthesis, as a secondary messenger, cAMP was a major contributor to the process of releasing follicle-stimulating hormone and luteinizing hormone. It was noteworthy that *RARA* participated in signaling pathways associated with reproduction, indicating its potential importance in regulating hormones. *RARA* was further predicted to be a transcription factor specifically involved in hair follicle morphogenesis at different stages [50]. We found a region with the highest *F*_ST _value (0.282032) on chromosome 8. *TNFAIP3*, the gene encoding A20, has been documented to be an anti-inflammatory and immune factor [51] and expressed increased abnormally in many types of tumor cells and tissues, which was closely associated with the progression, therapy and prognosis of cancer [52, 53]. Zammit [54] explored the impact of the *Tnfaip3* I325N variant on limiting fecundity by inducing hormonal imbalance, underscoring the role of the anti-inflammatory enzyme *TNFAIP3* in affecting fecundity. Naturally, further studies are needed to delve deeper into the specific mechanisms by which these genes influence sheep reproduction and the implications of mutations on fertility.

We also analyzed the potential selection signatures of HS vs. others using *F*_ST_ and XP-CLR methods, overlapping regions that *F*_ST_ > 0.25 and XP-CLR scores > 2.5 were selected as candidate regions. According to the reference genome annotation information, there are 39 genes located in these selected chromosome regions. We identified candidate genes associated with immune response (*BCAP29* [55], *ATP8B4* [56]), reproductive traits (*CC2D1B*) [57], teat number (*SYNDIG1L*) [58], and horn phenotype (*RXFP2*) [12]. The upregulation of critical genes during the follicular phase reflects the immune system’s role in follicular recruitment, while endocrine changes throughout the estrous cycle regulate immune gene expression, affecting follicular atresia and recruitment [59]. The immune system precisely regulates follicle development and maturation, influenced by the ovarian microenvironment [60]. The co-localization of *CC2D1B* and *DPY19L2* suggested that *CC2D1B* plays a crucial role in the reformation of the sperm nuclear envelope [57]. Li found patient P38 with pyriform-headed sperm showed a *CC2D1B* mutation, elucidating the significant role of *CC2D1B* in sperm-head formation [61]. In the present study, *CC2D1B* was located in a significant selective region (Fig. 2B, C), and the genotype pattern differed between the HS and other sheep breeds, suggesting that *CC2D1B* may play an important role in sheep fertility (Fig. 2F). Luongo [62] reported a case where a man exhibited total sperm immotility due to the *CCDC103* p.His154Pro mutation, which confirmed that *CCDC103* does indeed have an impact on sperm development. The *VRTN* gene variants exhibited a strong correlation with the number of thoracic vertebrae (TVN) in pigs [63]. The loss-of-function mutations of *CC2D1B* contributed to morphological abnormalities of the sperm head [61]. *SYNDIG1L* has been identified as a candidate gene for teat number in pigs [58]. In a study conducted by Lu et al., it was discovered that *C1QL1* played a crucial role in the regulation of follicle failure in the ovaries. This regulation occurred through a multidimensional collaborative mechanism involving intraovarian and endocrine control. These findings demonstrated that the loss of *C1QL1* led to the failure of ovarian follicles, disrupting their proper development and function [64].

A previous study has revealed a close relationship between the *FecB* gene and ovulation number in sheep [65]. Based on different *FecB* genotypes in Hu sheep, the *F*_ST_ analysis has identified *BMPR1B, PPM1K, UNC5C*, and *ABCG2* genes (Fig. 3A, B). It is widely known, *BMPR1B* plays a crucial role in the multiple births effect in sheep [66]. Studies using a *PPM1K*-deficient mouse model and downregulated *PPM1K* in human ovarian granulosa cells have shown that *PPM1K* deficiency results in impaired metabolism of branched-chain amino acids, contributing to polycystic ovary syndrome in females [67]. *ABCG2* was found to be associated with milk production in sheep [68].

Using single-lamb HS as the control group, we investigated the positively selected regions associated with multi-lambs, and then discovered genes related to sperm development. Research has shown that the removal of *CCDC63* leads to infertility in male mice, primarily due to the shortening of flagella. Although *CCDC63* did not participate in the formation of the outer dynein arms, it played a crucial role in sperm production [69]. Male mice lacking both PP1γ1 and PP1γ2 due to *PPP1CC* knockout were sterile because of impaired sperm morphogenesis. However, fertility and normal sperm function can be restored by introducing transgenic expression of PP1γ2 alone in the testis of *PPP1CC* mice [70]. *HVCN1* channels were associated with the cryotolerance of mammalian sperm [71] and played a crucial role in modulating sperm motility, kinematics, and facilitating calcium entry into the sperm head in pigs [72].

In conclusion, our study revealed significant genetic differentiations among six distinct sheep breeds at the whole-genome level. Additionally, we identified a set of genes associated with reproductive performance in Hu sheep and visualized the variations in these genes across different sheep breeds. These findings will contribute to the future identification of candidate genes related to reproduction in sheep and identify high-yield gene variants with greater precision which benefits breeding strategies.

### Electronic supplementary material

Below is the link to the electronic supplementary material.

Supplementary Material 1

Supplementary Material 2

Supplementary Material 3

Supplementary Material 4

Supplementary Material 5

Supplementary Material 6

Supplementary Material 7

Supplementary Material 8

Supplementary Material 9

Supplementary Material 10

Supplementary Material 11

## Data Availability

The datasets generated during the present study are available in the NCBI under BioProject accession number PRJNA1058981.

## Abbreviations

SNP: Single nucleotide polymorphism
*Ho*: Observed heterozygosity
*He*: Expected heterozygosity
*π*: Nucleotide diversity
ROH: Runs of homozygosity
LD: Linkage disequilibrium
KEGG: Kyoto encyclopaedia of genes and genomes
*F*_ST_: Fixation index
XP-CLR: Cross-population composite likelihood ratio test
PCA: Principal component analysis
