# Supplementary material for: Comparative whole-genome resequencing to uncover selection signatures linked to litter size in Hu Sheep and five other breeds
Source: BMC Genomics. 2024 May 15;25:480. doi: 10.1186/s12864-024-10396-x (PMC11094944; doi:10.1186/s12864-024-10396-x)
Supplement: Supplementary file 1 — Supplementary Material 1 [file 12864_2024_10396_MOESM1_ESM.docx]

**Supplementary Table 1.** Quality of sheep sequencing data.

| Population | Individual ID | Raw bases | Raw reads | Clean bases | Clean reads | Clean bases rate | Clean reads rate | Q30 | GC | Duplication | Average Sequencing depth |
| --- | --- | --- | --- | --- | --- | --- | --- | --- | --- | --- | --- |
| Hu Sheep (HS) | H1 | 37203000000 | 248018552 | 37172000000 | 247907576 | 0.999 | 1 | 0.914 | 0.433 | 0.073 | 11.1863 |
|  | H2 | 31608000000 | 210721486 | 31587000000 | 210634586 | 0.999 | 1 | 0.899 | 0.431 | 0.062 | 9.51659 |
|  | H3 | 33059000000 | 220395860 | 33034000000 | 220301094 | 0.999 | 1 | 0.906 | 0.434 | 0.068 | 9.84879 |
|  | H4 | 32636000000 | 217576202 | 32615000000 | 217484690 | 0.999 | 1 | 0.908 | 0.43 | 0.072 | 9.78878 |
|  | H5 | 38745000000 | 258301984 | 38716000000 | 258189928 | 0.999 | 1 | 0.908 | 0.431 | 0.079 | 11.4472 |
|  | H6 | 31412000000 | 209416368 | 31389000000 | 209327642 | 0.999 | 1 | 0.89 | 0.434 | 0.065 | 9.23411 |
|  | H7 | 31741000000 | 211608948 | 31718000000 | 211516246 | 0.999 | 1 | 0.913 | 0.434 | 0.068 | 9.51051 |
|  | H8 | 38119000000 | 254129248 | 38086000000 | 254021464 | 0.999 | 1 | 0.914 | 0.431 | 0.076 | 11.4251 |
|  | H9 | 30033000000 | 200219176 | 30012000000 | 200135356 | 0.999 | 1 | 0.874 | 0.434 | 0.061 | 8.68437 |
|  | H10 | 38164000000 | 254425626 | 38137000000 | 254315524 | 0.999 | 1 | 0.899 | 0.434 | 0.073 | 11.2372 |
|  | H11 | 39937000000 | 266248856 | 39909000000 | 266136416 | 0.999 | 1 | 0.912 | 0.433 | 0.079 | 11.8811 |
|  | H12 | 46559000000 | 310390228 | 46522000000 | 310258052 | 0.999 | 1 | 0.898 | 0.436 | 0.08 | 13.4484 |
|  | H13 | 37257000000 | 248379114 | 37231000000 | 248278072 | 0.999 | 1 | 0.894 | 0.431 | 0.073 | 11.0445 |
|  | H14 | 32317000000 | 215446694 | 32292000000 | 215351602 | 0.999 | 1 | 0.908 | 0.431 | 0.068 | 9.73907 |
|  | H15 | 35882000000 | 239214324 | 35853000000 | 239101616 | 0.999 | 1 | 0.926 | 0.432 | 0.079 | 10.7491 |
|  | H16 | 36724000000 | 244829406 | 36696000000 | 244717176 | 0.999 | 1 | 0.917 | 0.433 | 0.079 | 10.9345 |
|  | H17 | 31443000000 | 209620446 | 31418000000 | 209522130 | 0.999 | 1 | 0.923 | 0.431 | 0.062 | 9.61928 |
|  | H18 | 36382000000 | 242546520 | 36354000000 | 242438974 | 0.999 | 1 | 0.906 | 0.434 | 0.075 | 10.7137 |
|  | H19 | 35917000000 | 239445312 | 35890000000 | 239340700 | 0.999 | 1 | 0.901 | 0.433 | 0.074 | 10.6007 |
|  | H20 | 31449000000 | 209663154 | 31428000000 | 209568202 | 0.999 | 1 | 0.916 | 0.43 | 0.067 | 9.55816 |
|  | H21 | 36519000000 | 243460666 | 36491000000 | 243350862 | 0.999 | 1 | 0.917 | 0.433 | 0.072 | 10.9634 |
|  | H22 | 36330000000 | 242199174 | 36301000000 | 242093590 | 0.999 | 1 | 0.896 | 0.436 | 0.076 | 10.5401 |
|  | H23 | 31140000000 | 207600646 | 30624000000 | 204228728 | 0.983 | 0.984 | 0.921 | 0.433 | 0.042 | 9.68456 |
|  | H24 | 39125000000 | 260830244 | 39092000000 | 260711764 | 0.999 | 1 | 0.914 | 0.434 | 0.078 | 11.6027 |
|  | H25 | 31154000000 | 207694868 | 31120000000 | 207531974 | 0.999 | 0.999 | 0.915 | 0.434 | 0.158 | 8.46064 |
|  | H26 | 33776000000 | 225170040 | 33750000000 | 225069086 | 0.999 | 1 | 0.921 | 0.431 | 0.07 | 10.2848 |
|  | H27 | 33408000000 | 222720766 | 33381000000 | 222619564 | 0.999 | 1 | 0.918 | 0.431 | 0.076 | 9.99565 |
|  | H28 | 31509000000 | 210060842 | 31487000000 | 209969164 | 0.999 | 1 | 0.911 | 0.431 | 0.066 | 9.52517 |
| Liangshan Black Sheep (LB) | L8 | 35631000000 | 237542362 | 35601000000 | 237433418 | 0.999 | 1 | 0.924 | 0.432 | 0.087 | 10.6632 |
|  | L13 | 39160000000 | 261065560 | 39130000000 | 260957286 | 0.999 | 1 | 0.915 | 0.439 | 0.054 | 11.9456 |
|  | L14 | 33216000000 | 221440840 | 33190000000 | 221350592 | 0.999 | 1 | 0.878 | 0.443 | 0.061 | 9.59786 |
|  | L16 | 30305000000 | 202033800 | 30278000000 | 201945346 | 0.999 | 1 | 0.903 | 0.44 | 0.062 | 9.15698 |
|  | L17 | 31085000000 | 207230478 | 31060000000 | 207139054 | 0.999 | 1 | 0.895 | 0.439 | 0.078 | 8.96946 |
|  | L18 | 32854000000 | 219029220 | 32830000000 | 218931328 | 0.999 | 1 | 0.916 | 0.434 | 0.074 | 9.90117 |
|  | L20 | 32338000000 | 215589010 | 31807000000 | 212106082 | 0.984 | 0.984 | 0.924 | 0.436 | 0.047 | 9.90136 |
|  | L22 | 30412000000 | 202743988 | 29914000000 | 199497390 | 0.984 | 0.984 | 0.916 | 0.432 | 0.047 | 9.28997 |
|  | L23 | 33596000000 | 223976322 | 33038000000 | 220325810 | 0.983 | 0.984 | 0.92 | 0.436 | 0.044 | 10.466 |
|  | L24 | 30770000000 | 205131250 | 30287000000 | 201999062 | 0.984 | 0.985 | 0.889 | 0.44 | 0.042 | 9.33763 |
|  | L30 | 37422000000 | 249482052 | 36833000000 | 245645000 | 0.984 | 0.985 | 0.899 | 0.44 | 0.047 | 11.1328 |
|  | L31 | 36735000000 | 244901252 | 36130000000 | 240975050 | 0.984 | 0.984 | 0.912 | 0.441 | 0.048 | 10.9804 |
|  | L33 | 37948000000 | 252986534 | 37320000000 | 248886796 | 0.983 | 0.984 | 0.918 | 0.436 | 0.046 | 11.5263 |
|  | L42 | 34148000000 | 227655840 | 33597000000 | 224060770 | 0.984 | 0.984 | 0.911 | 0.435 | 0.042 | 10.4576 |
|  | L44 | 33681000000 | 224537346 | 33140000000 | 221004480 | 0.984 | 0.984 | 0.906 | 0.439 | 0.046 | 10.2003 |
|  | L48 | 33063000000 | 220417812 | 32522000000 | 216895532 | 0.984 | 0.984 | 0.915 | 0.436 | 0.047 | 10.1545 |
|  | L49 | 32285000000 | 215231104 | 31757000000 | 211801456 | 0.984 | 0.984 | 0.907 | 0.435 | 0.049 | 9.74148 |
|  | L52 | 32128000000 | 214188680 | 31611000000 | 210820876 | 0.984 | 0.984 | 0.905 | 0.442 | 0.044 | 9.65483 |
|  | L53 | 33593000000 | 223953196 | 33570000000 | 223863370 | 0.999 | 1 | 0.884 | 0.434 | 0.05 | 10.1418 |
| White Xizang Sheep (WX) | BZ3 | 20866000000 | 166927230 | 19724000000 | 158123772 | 0.945 | 0.947 | 0.847 | 0.431 | 0.093 | 5.79049 |
|  | BZ5 | 20059000000 | 160468174 | 19106000000 | 153245978 | 0.952 | 0.955 | 0.884 | 0.432 | 0.081 | 5.65469 |
|  | BZ7 | 20314000000 | 162515200 | 19380000000 | 155484106 | 0.954 | 0.957 | 0.886 | 0.433 | 0.093 | 5.4762 |
|  | BZ12 | 19827000000 | 158617274 | 18762000000 | 150519664 | 0.946 | 0.949 | 0.871 | 0.434 | 0.076 | 5.38699 |
|  | BZ14 | 15372000000 | 122975142 | 15292000000 | 122377556 | 0.995 | 0.995 | 0.906 | 0.422 | 0.009 | 4.96169 |
|  | BZ16 | 24224000000 | 193794308 | 22827000000 | 183088296 | 0.942 | 0.945 | 0.862 | 0.432 | 0.085 | 6.25225 |
|  | BZ19 | 24078000000 | 192621228 | 22895000000 | 183683774 | 0.951 | 0.954 | 0.886 | 0.433 | 0.093 | 6.32855 |
|  | BZ23 | 23320000000 | 186557454 | 22535000000 | 180824206 | 0.966 | 0.969 | 0.899 | 0.434 | 0.105 | 6.325 |
|  | BZ26 | 17623000000 | 140983442 | 16926000000 | 135745782 | 0.96 | 0.963 | 0.892 | 0.433 | 0.095 | 4.83289 |
|  | BZ34 | 16225000000 | 129799556 | 15785000000 | 126544646 | 0.973 | 0.975 | 0.907 | 0.433 | 0.075 | 4.69518 |
| Oula Sheep (OL) | OL14 | 23954000000 | 191632022 | 23208000000 | 186109438 | 0.969 | 0.971 | 0.906 | 0.434 | 0.096 | 6.6801 |
|  | OL15 | 26942000000 | 215533296 | 25828000000 | 207196706 | 0.959 | 0.961 | 0.885 | 0.437 | 0.186 | 6.58192 |
|  | OL21 | 21810000000 | 174480246 | 20859000000 | 167283770 | 0.956 | 0.959 | 0.882 | 0.432 | 0.112 | 5.90242 |
|  | OL23 | 25261000000 | 202089042 | 24451000000 | 196126020 | 0.968 | 0.97 | 0.905 | 0.435 | 0.1 | 6.99054 |
|  | OL27 | 16976000000 | 135811646 | 16885000000 | 135155894 | 0.995 | 0.995 | 0.892 | 0.423 | 0.008 | 5.47638 |
|  | OL31 | 14664000000 | 117314570 | 14578000000 | 116659534 | 0.994 | 0.994 | 0.897 | 0.424 | 0.009 | 4.75731 |
|  | OL33 | 17976000000 | 143805422 | 17307000000 | 138777560 | 0.963 | 0.965 | 0.899 | 0.433 | 0.08 | 5.13036 |
|  | OL40 | 24968000000 | 199747894 | 23904000000 | 191614430 | 0.957 | 0.959 | 0.883 | 0.43 | 0.112 | 6.78156 |
|  | OL41 | 21996000000 | 175971634 | 21161000000 | 169692876 | 0.962 | 0.964 | 0.895 | 0.432 | 0.095 | 5.6782 |
| Bamei Mutton Sheep (BM) | SRR9990327 | 17749000000 | 140866972 | 17305000000 | 137377496 | 0.975 | 0.975 | 0.876 | 0.432 | 0.003 | 5.83635 |
|  | SRR9990328 | 16295000000 | 129323244 | 15975000000 | 126819444 | 0.98 | 0.981 | 0.897 | 0.438 | 0.009 | 5.34837 |
|  | SRR9990329 | 18959000000 | 150471712 | 18588000000 | 147573316 | 0.98 | 0.981 | 0.905 | 0.441 | 0.007 | 6.12884 |
|  | SRR9990330 | 16095000000 | 127735804 | 15132000000 | 120117440 | 0.94 | 0.94 | 0.93 | 0.456 | 0.003 | 5.19923 |
|  | SRR9990331 | 18666000000 | 148142476 | 18214000000 | 144595358 | 0.976 | 0.976 | 0.879 | 0.433 | 0.004 | 6.0642 |
|  | SRR9990332 | 18357000000 | 145687552 | 17928000000 | 142312002 | 0.977 | 0.977 | 0.879 | 0.432 | 0.006 | 6.03667 |
|  | SRR9990333 | 18989000000 | 150706876 | 18392000000 | 146010166 | 0.969 | 0.969 | 0.877 | 0.464 | 0.003 | 6.2338 |
|  | SRR9990334 | 17402000000 | 138109722 | 16982000000 | 134820544 | 0.976 | 0.976 | 0.876 | 0.43 | 0.004 | 5.69816 |
|  | SRR9990335 | 18053000000 | 143280386 | 16954000000 | 134582386 | 0.939 | 0.939 | 0.93 | 0.444 | 0.003 | 5.84681 |
|  | SRR9990336 | 19597000000 | 155532926 | 17816000000 | 141421052 | 0.909 | 0.909 | 0.918 | 0.429 | 0.004 | 6.01665 |
| Poll Dorset Sheep (PD) | TST4 | 18501000000 | 148006044 | 17554000000 | 140798580 | 0.949 | 0.951 | 0.882 | 0.434 | 0.066 | 5.01404 |
|  | TST12 | 21437000000 | 171494096 | 20458000000 | 164002052 | 0.954 | 0.956 | 0.879 | 0.43 | 0.147 | 5.52505 |
|  | TST20 | 17553000000 | 140422528 | 16766000000 | 134392842 | 0.955 | 0.957 | 0.887 | 0.431 | 0.062 | 5.15355 |
|  | TST27 | 18430000000 | 147437304 | 17498000000 | 140187246 | 0.949 | 0.951 | 0.872 | 0.428 | 0.107 | 5.01137 |
|  | TST41 | 14369000000 | 114954276 | 13878000000 | 111189514 | 0.966 | 0.967 | 0.897 | 0.435 | 0.102 | 3.92987 |
|  | TST43 | 27287000000 | 218296022 | 25956000000 | 208028010 | 0.951 | 0.953 | 0.875 | 0.433 | 0.115 | 7.17129 |
|  | TST48 | 22558000000 | 180467444 | 21607000000 | 173221744 | 0.958 | 0.96 | 0.889 | 0.433 | 0.075 | 6.32405 |
|  | TST50 | 20776000000 | 166207510 | 19979000000 | 160138386 | 0.962 | 0.963 | 0.887 | 0.431 | 0.112 | 5.59788 |
|  | TST52 | 24138000000 | 193105256 | 22997000000 | 184285564 | 0.953 | 0.954 | 0.885 | 0.429 | 0.068 | 6.93594 |
|  | TST56 | 20898000000 | 167185806 | 20045000000 | 160608002 | 0.959 | 0.961 | 0.884 | 0.428 | 0.112 | 5.73166 |
