# Supplementary material for: Comparative whole-genome resequencing to uncover selection signatures linked to litter size in Hu Sheep and five other breeds
Source: BMC Genomics. 2024 May 15;25:480. doi: 10.1186/s12864-024-10396-x (PMC11094944; doi:10.1186/s12864-024-10396-x)
Supplement: Supplementary file 2 — Supplementary Material 2 [file 12864_2024_10396_MOESM2_ESM.docx]

**Supplementary Table 2.** Putatively selected region in six breeds by global-F_ST_.

| Chromosome | Position | | Gene name |
| --- | --- | --- | --- |
|  | Start | End |  |
| 1 | 29360001 | 29480000 | *CZIB, MAGOH, LRP8* |
| 1 | 29680001 | 29760000 | *GLIS1* |
| 1 | 110840001 | 111080000 | *IL6R, TDRD10, CHRNB2, SHE, ADAR* |
| 1 | 248160001 | 248280000 | *U6* |
| 1 | 256360001 | 256440000 | *MBNL1* |
| 2 | 78560001 | 78640000 | *MIR101-2, RCL1* |
| 2 | 79800001 | 79880000 | *IL33* |
| 2 | 150080001 | 150160000 | *MYO3B* |
| 2 | 159280001 | 159360000 | *U6, GCA, KCNH7* |
| 3 | 139320001 | 139520000 | *CRADD* |
| 3 | 141960001 | 142120000 | *HOXC5, HOXC6, Hoxc8, HOXC9, MIR196A2, HOXC11, HOXC4, HOXC10, HOXC12, HOXC13* |
| 3 | 143280001 | 143400000 | *KRT73, KRT74, KRT71, KRT5, KRT72* |
| 3 | 150800001 | 150880000 | *ARID2* |
| 3 | 157680001 | 157760000 | *LRRK2* |
| 3 | 173240001 | 173320000 | *ATP23* |
| 3 | 173800001 | 173880000 | *ARHGAP9, INHBE, INHBC, GLI1, R3HDM2* |
| 3 | 181880001 | 181960000 | *U6* |
| 3 | 182920001 | 183000000 | *ARL1, UTP20* |
| 3 | 220600001 | 220760000 | *KLRF1, CD69* |
| 4 | 35600001 | 35680000 | *IGF2BP3* |
| 4 | 52600001 | 52720000 | *PUS7, RINT1, EFCAB10, ATXN7L1* |
| 4 | 74920001 | 75000000 | *JAZF1* |
| 4 | 75640001 | 75800000 | *EVX1, HOXA13, HOXA11, MIR196B, HOXA7, HOXA6, HOXA5, HOXA4, HOXA9* |
| 5 | 116800001 | 117080000 | *WDR36* |
| 6 | 98680001 | 98760000 | *CXCL6* |
| 6 | 129400001 | 129840000 | *DGKQ, ATP5ME, TMEM271, TMEM175, PCGF3, PDE6B, PIGG, CPLX1, SLC49A3* |
| 7 | 37000001 | 37080000 | *U6, MGA* |
| 7 | 37800001 | 37920000 | *LRRC57, ZNF106, HAUS2, SNAP23* |
| 7 | 47240001 | 47360000 | *HERC1* |
| 7 | 61880001 | 62000000 | *ATP8B4* |
| 7 | 62280001 | 62360000 | *U6* |
| 7 | 96760001 | 96960000 | *SNORA79, GTF2A1, TSHR* |
| 8 | 35160001 | 35280000 | *PREP* |
| 8 | 35480001 | 35680000 | *BVES, LIN28B* |
| 8 | 69120001 | 69280000 | *TNFAIP3* |
| 10 | 2840001 | 2920000 | *TDRD3* |
| 10 | 30840001 | 31040000 | *RXFP2* |
| 11 | 22520001 | 22920000 | *RAPGEFL1, SNORD124, CSF3, PSMD3, LRRC3C, RARA, CDC6, WIPF2, CASC3, MSL1, NR1D1, MED24, GSDMA, TOP2A, THRA* |
| 11 | 26480001 | 26560000 | *KAT7* |
| 11 | 28880001 | 28960000 | *MIEF2, SMCR8, ALKBH5, LLGL1, FLII, TOP3A, PRPSAP2* |
| 11 | 36520001 | 36600000 | *CLEC10A, SLC16A11, BCL6B, MIR497, MIR195, RNASEK, U6, SLC16A13, C17orf49, ALOX12* |
| 11 | 44840001 | 44960000 | *NF1* |
| 13 | 25160001 | 25240000 | *OTUD1* |
| 13 | 53360001 | 53440000 | *PANK2* |
| 13 | 54720001 | 54840000 | *EBF4* |
| 13 | 56240001 | 56600000 | *ABHD16B, LIME1, ARFRP1, TNFRSF6B, STMN3, FNDC11, PTK6, CHRNA4, TPD52L2, ZBTB46, ZGPAT, GMEB2, SRMS, EEF1A2, ARFGAP1, RTEL1, KCNQ2* |
| 14 | 14600001 | 14720000 | *CYBA, RNF166, ZC3H18, IL17C, SNAI3, CTU2, PIEZO1, MVD* |
| 14 | 18120001 | 18200000 | *LONP2, SIAH1* |
| 14 | 54840001 | 54920000 | *TEX101, BSP5, BSP5L* |
| 14 | 59360001 | 59480000 | *SNORD33, SNORD34, SNORD35A, RPS11, SNORD35B, MIR150, FCGRT, NOSIP, RRAS, ALDH16A1, RCN3, PRRG2, PRR12, FLT3LG* |
| 15 | 23080001 | 23240000 | *ARHGAP20* |
| 15 | 42840001 | 42920000 | *TEAD1* |
| 15 | 51840001 | 52040000 | *HBB* |
| 16 | 34480001 | 34560000 | *OXCT1* |
| 16 | 41200001 | 41280000 | *PRLR* |
| 18 | 68160001 | 68240000 | *CDC42BPB* |
| 19 | 33240001 | 33480000 | *MITF* |
| 19 | 53560001 | 53680000 | *CATHL3, BAC5, SC5, CDC25A* |
| 19 | 54920001 | 55040000 | *CCRL2, CCR5, CCR2, LTF* |
| 20 | 44880001 | 44960000 | *U6, JARID2* |
| 20 | 54040001 | 54120000 | *RIPK1* |
| 20 | 54680001 | 54920000 | *GMDS* |
| 21 | 41680001 | 41760000 | *TKFC, TMEM138, DDB1, CYB561A3* |
| 21 | 43680001 | 43760000 | *LGALS12* |
| 21 | 46840001 | 46960000 | *GRK2, KDM2A* |
| 22 | 10760001 | 10880000 | *PAPSS2, ATAD1* |
| 23 | 27360001 | 27440000 | *NOL4* |
| 25 | 6920001 | 7040000 | *TARBP1* |
| 25 | 7040001 | 7160000 | *IRF2BP2, 5S_rRNA* |
| 26 | 39440001 | 39560000 | *SNORA70* |
