# Supplementary material for: Comparative whole-genome resequencing to uncover selection signatures linked to litter size in Hu Sheep and five other breeds
Source: BMC Genomics. 2024 May 15;25:480. doi: 10.1186/s12864-024-10396-x (PMC11094944; doi:10.1186/s12864-024-10396-x)
Supplement: Supplementary file 3 — Supplementary Material 3 [file 12864_2024_10396_MOESM3_ESM.docx]

**Supplementary Table 3.** KEGG Analysis for the candidate genes in the six sheep breeds.

| Term | P-Value | Corrected P-Value | Input |
| --- | --- | --- | --- |
| Cytokine-cytokine receptor interaction | 0.00282559 | 0.35319926 | *CCR5, IL17C, IL6R, INHBC, INHBE, CSF3, TNFRSF6B, IL33* |
| NOD-like receptor signaling pathway | 0.00855521 | 0.39350255 | *RIPK1, SC5, BAC5, CATHL3, TNFAIP3* |
| Staphylococcus aureus infection | 0.01297373 | 0.39350255 | *SC5, BAC5, CATHL3* |
| Cytosolic DNA-sensing pathway | 0.01392297 | 0.39350255 | *RIPK1, ADAR, IL33* |
| Signaling pathways regulating pluripotency of stem cells | 0.0157401 | 0.39350255 | *PCGF3, INHBC, JARID2, INHBE* |
| Fructose and mannose metabolism | 0.02414181 | 0.42056023 | *GMDS, TKFC* |
| IL-17 signaling pathway | 0.02421473 | 0.42056023 | *IL17C, TNFAIP3, CSF3* |
| Salivary secretion | 0.02980566 | 0.42056023 | *SC5, BAC5, CATHL3* |
| Nicotine addiction | 0.03191585 | 0.42056023 | *CHRNA4, CHRNB2* |
| Transcriptional misregulation in cancer | 0.0376584 | 0.42056023 | *RARA, MITF, HOXA9, HOXA11* |
| Cholinergic synapse | 0.03850627 | 0.42056023 | *CHRNA4, CHRNB2, KCNQ2* |
| Hedgehog signaling pathway | 0.04357613 | 0.42056023 | *GLI1, GRK2* |
| Hematopoietic cell lineage | 0.04373826 | 0.42056023 | *IL6R, FLT3LG, CSF3* |
| Malaria | 0.05486635 | 0.48987813 | *HBB, CSF3* |
| Sulfur relay system | 0.06319031 | 0.50737839 | *CTU2* |
| Glycerolipid metabolism | 0.06705647 | 0.50737839 | *DGKQ, TKFC* |
| Sulfur metabolism | 0.06928668 | 0.50737839 | *PAPSS2* |
| Mitophagy - animal | 0.07438677 | 0.50737839 | *RRAS, MITF* |
| Ubiquitin mediated proteolysis | 0.07712152 | 0.50737839 | *HERC1, DDB1, SIAH1* |
| Synthesis and degradation of ketone bodies | 0.08136135 | 0.50850842 | *OXCT1* |
| Neuroactive ligand-receptor interaction | 0.10047084 | 0.54817704 | *TSHR, CHRNB2, RXFP2, CHRNA4, THRA* |
| Endocytosis | 0.10323689 | 0.54817704 | *WIPF2, GRK2, CCR5, ARFGAP1* |
| EGFR tyrosine kinase inhibitor resistance | 0.1059645 | 0.54817704 | *NF1, IL6R* |
| RNA transport | 0.11447878 | 0.54817704 | *CASC3, EEF1A2, MAGOH* |
| Ribosome biogenesis in eukaryotes | 0.12073374 | 0.54817704 | *WDR36, RCL1* |
| Pantothenate and CoA biosynthesis | 0.1224091 | 0.54817704 | *PANK2* |
| TGF-beta signaling pathway | 0.12721116 | 0.54817704 | *INHBC, INHBE* |
| Selenocompound metabolism | 0.12812224 | 0.54817704 | *PAPSS2* |
| Viral protein interaction with cytokine and cytokine receptor | 0.12938843 | 0.54817704 | *CCR5, IL6R* |
| Necroptosis | 0.13160402 | 0.54817704 | *RIPK1, IL33, TNFAIP3* |
| Terpenoid backbone biosynthesis | 0.13943781 | 0.54817704 | *MVD* |
| mRNA surveillance pathway | 0.14040154 | 0.54817704 | *CASC3, MAGOH* |
| NF-kappa B signaling pathway | 0.15160784 | 0.54817704 | *RIPK1, TNFAIP3* |
| Renin-angiotensin system | 0.16163277 | 0.54817704 | *PREP* |
| Glycosylphosphatidylinositol (GPI)-anchor biosynthesis | 0.16163277 | 0.54817704 | *PIGG* |
| Tuberculosis | 0.16509444 | 0.54817704 | *SC5, BAC5, CATHL3* |
| Phototransduction | 0.17251587 | 0.54817704 | *PDE6B* |
| Butanoate metabolism | 0.17790463 | 0.54817704 | *OXCT1* |
| Hippo signaling pathway - multiple species | 0.18325849 | 0.54817704 | *TEAD1* |
| RIG-I-like receptor signaling pathway | 0.18381764 | 0.54817704 | *RIPK1, TKFC* |
| Th17 cell differentiation | 0.18850003 | 0.54817704 | *RARA, IL6R* |
| Circadian rhythm | 0.18857769 | 0.54817704 | *NR1D1* |
| Thyroid hormone signaling pathway | 0.19084746 | 0.54817704 | *MED24, THRA* |
| TNF signaling pathway | 0.20738187 | 0.54817704 | *RIPK1, TNFAIP3* |
| SNARE interactions in vesicular transport | 0.20951225 | 0.54817704 | *SNAP23* |
| Cell cycle | 0.20975683 | 0.54817704 | *CDC25A, CDC6* |
| cAMP signaling pathway | 0.21419313 | 0.54817704 | *RRAS, TSHR, GLI1* |
| Ras signaling pathway | 0.21419313 | 0.54817704 | *RRAS, FLT3LG, NF1* |
| Viral carcinogenesis | 0.21757543 | 0.54817704 | *DDB1, GTF2A1, CCR5* |
| Epstein-Barr virus infection | 0.21927082 | 0.54817704 | *RIPK1, PSMD3, TNFAIP3* |
| Human immunodeficiency virus 1 infection | 0.22607939 | 0.55193806 | *RIPK1, DDB1, CCR5* |
| African trypanosomiasis | 0.22990972 | 0.55193806 | *HBB* |
| Human cytomegalovirus infection | 0.23636775 | 0.55193806 | *RIPK1, CCR5, IL6R* |
| Purine metabolism | 0.23843724 | 0.55193806 | *PDE6B, PAPSS2* |
| Homologous recombination | 0.24978382 | 0.56769049 | *TOP3A* |
| Phospholipase D signaling pathway | 0.25526634 | 0.56914206 | *RRAS, DGKQ* |
| Proteasome | 0.25952878 | 0.56914206 | *PSMD3* |
| Nucleotide excision repair | 0.26435396 | 0.56972837 | *DDB1* |
| Basal transcription factors | 0.27391074 | 0.57044549 | *GTF2A1* |
| Hippo signaling pathway | 0.27934066 | 0.57044549 | *TEAD1, LLGL1* |
| Antifolate resistance | 0.29265553 | 0.57044549 | *ALOX12* |
| Fanconi anemia pathway | 0.29265553 | 0.57044549 | *TOP3A* |
| Valine, leucine and isoleucine degradation | 0.29265553 | 0.57044549 | *OXCT1* |
| Pathways in cancer | 0.29575154 | 0.57044549 | *RARA, MITF, GLI1, FLT3LG, IL6R* |
| Amino sugar and nucleotide sugar metabolism | 0.3018467 | 0.57044549 | *GMDS* |
| Measles | 0.30336295 | 0.57044549 | *ADAR, TNFAIP3* |
| Cellular senescence | 0.30575879 | 0.57044549 | *RRAS, CDC25A* |
| Regulation of lipolysis in adipocytes | 0.31091911 | 0.57154249 | *TSHR* |
| MAPK signaling pathway | 0.32428966 | 0.58748127 | *RRAS, FLT3LG, NF1* |
| Tight junction | 0.33436902 | 0.59708754 | *BVES, LLGL1* |
| Chemokine signaling pathway | 0.35090635 | 0.61308092 | *GRK2, CCR5* |
| Basal cell carcinoma | 0.35455264 | 0.61308092 | *GLI1* |
| Acute myeloid leukemia | 0.35876192 | 0.61308092 | *RARA* |
| Legionellosis | 0.3629439 | 0.61308092 | *EEF1A2* |
| MicroRNAs in cancer | 0.37428221 | 0.61450409 | *CDC25A, MIR150* |
| Influenza A | 0.37428221 | 0.61450409 | *ADAR, IL33* |
| Melanoma | 0.37940242 | 0.61450409 | *MITF* |
| Thyroid hormone synthesis | 0.38345055 | 0.61450409 | *TSHR* |
| Arachidonic acid metabolism | 0.39146822 | 0.61787203 | *ALOX12* |
| Autoimmune thyroid disease | 0.3954381 | 0.61787203 | *TSHR* |
| Synaptic vesicle cycle | 0.41106175 | 0.63435456 | *CPLX1* |
| p53 signaling pathway | 0.42251544 | 0.64407842 | *SIAH1* |
| Platinum drug resistance | 0.44111482 | 0.66432955 | *TOP2A* |
| Progesterone-mediated oocyte maturation | 0.45198728 | 0.66948052 | *CDC25A* |
| Jak-STAT signaling pathway | 0.45524676 | 0.66948052 | *IL6R, CSF3* |
| Morphine addiction | 0.46264942 | 0.67245555 | *GRK2* |
| PI3K-Akt signaling pathway | 0.48536007 | 0.67776647 | *IL6R, FLT3LG, CSF3* |
| Choline metabolism in cancer | 0.48673242 | 0.67776647 | *DGKQ* |
| Phosphatidylinositol signaling system | 0.48673242 | 0.67776647 | *DGKQ* |
| Glycerophospholipid metabolism | 0.49008409 | 0.67776647 | *DGKQ* |
| Melanogenesis | 0.49341399 | 0.67776647 | *MITF* |
| Inflammatory mediator regulation of TRP channels | 0.50327454 | 0.68379694 | *ALOX12* |
| Glutamatergic synapse | 0.51294424 | 0.68430552 | *GRK2* |
| C-type lectin receptor signaling pathway | 0.52242677 | 0.68430552 | *RRAS* |
| Serotonergic synapse | 0.52554664 | 0.68430552 | *ALOX12* |
| HIF-1 signaling pathway | 0.52554664 | 0.68430552 | *IL6R* |
| Toll-like receptor signaling pathway | 0.54384484 | 0.70083099 | *RIPK1* |
| Metabolic pathways | 0.55686535 | 0.70192502 | *PAPSS2, PANK2, DGKQ, TKFC, PDE6B, GMDS, OXCT1, ALOX12, MVD, ATP5ME* |
| Platelet activation | 0.56715541 | 0.70192502 | *SNAP23* |
| Toxoplasmosis | 0.56715541 | 0.70192502 | *CCR5* |
| Carbon metabolism | 0.56715541 | 0.70192502 | *TKFC* |
| Autophagy - animal | 0.58385754 | 0.70434591 | *RRAS* |
| Osteoclast differentiation | 0.58657824 | 0.70434591 | *MITF* |
| Relaxin signaling pathway | 0.58928126 | 0.70434591 | *RXFP2* |
| Estrogen signaling pathway | 0.59728533 | 0.70434591 | *RARA* |
| Apelin signaling pathway | 0.59728533 | 0.70434591 | *RRAS* |
| Yersinia infection | 0.6051343 | 0.70693259 | *WIPF2* |
| Oxidative phosphorylation | 0.63021528 | 0.72578466 | *ATP5ME* |
| Apoptosis | 0.63742594 | 0.72578466 | *RIPK1* |
| Spliceosome | 0.63979828 | 0.72578466 | *MAGOH* |
| Parkinson disease | 0.64449678 | 0.72578466 | *LRRK2* |
| Non-alcoholic fatty liver disease (NAFLD) | 0.66046675 | 0.73712807 | *IL6R* |
| Wnt signaling pathway | 0.66927005 | 0.74034298 | *SIAH1* |
| Hepatitis C | 0.69029799 | 0.75675753 | *RIPK1* |
| Hepatitis B | 0.70030794 | 0.75675753 | *DDB1* |
| Hepatocellular carcinoma | 0.70227099 | 0.75675753 | *ARID2* |
| Axon guidance | 0.71565895 | 0.7645929 | *RRAS* |
| Proteoglycans in cancer | 0.75717627 | 0.78872325 | *RRAS* |
| Rap1 signaling pathway | 0.75717627 | 0.78872325 | *RRAS* |
| Kaposi sarcoma-associated herpesvirus infection | 0.76035082 | 0.78872325 | *CCR5* |
| Regulation of actin cytoskeleton | 0.76348411 | 0.78872325 | *RRAS* |
| Thermogenesis | 0.80841931 | 0.82829848 | *ATP5ME* |
| Ribosome | 0.81705547 | 0.83034092 | *RPS11* |
| Human papillomavirus infection | 0.91608675 | 0.92347454 | *LLGL1* |
| Olfactory transduction | 0.99945891 | 0.99945891 | *GRK2* |
