# Supplementary material for: Comparative whole-genome resequencing to uncover selection signatures linked to litter size in Hu Sheep and five other breeds
Source: BMC Genomics. 2024 May 15;25:480. doi: 10.1186/s12864-024-10396-x (PMC11094944; doi:10.1186/s12864-024-10396-x)
Supplement: Supplementary file 4 — Supplementary Material 4 [file 12864_2024_10396_MOESM4_ESM.docx]

**Supplementary Table 4.** Putatively selected region selected by XP-CLR and *F*_ST_ in HS vs. others.

| No. | Chromosome | Position | | Gene name |
| --- | --- | --- | --- | --- |
|  |  | Start | End |  |
| 1 | 1 | 28320001 | 28400000 | *ZFYVE9, ORC1, CC2D1B* |
| 2 | 1 | 110840001 | 110960000 | *TDRD10, SHE, IL6R* |
| 3 | 1 | 111000001 | 111080000 | *ADAR* |
| 4 | 2 | 255360001 | 255440000 | *WASF2, U6, MAP3K6,* *GPR3,* *CD164L2* |
| 5 | 3 | 139320001 | 139520000 | *CRADD* |
| 6 | 3 | 146280001 | 146400000 | *FAIM2* |
| 7 | 3 | 150720001 | 150880000 | *ARID2* |
| 8 | 3 | 220600001 | 220760000 | *KLRF1* |
| 9 | 4 | 54600001 | 54680000 | *SNORA70, BCAP29* |
| 10 | 4 | 75600001 | 75800000 | *MIR196B, HOXA9, HOXA7, HOXA6, HOXA5, HOXA4, HOXA13, HOXA11* |
| 11 | 7 | 89240001 | 89320000 | *LIN52* |
| 12 | 7 | 89320001 | 89480000 | *VSX2, VRTN, ABCD4* |
| 13 | 10 | 30920001 | 31040000 | *RXFP2* |
| 14 | 10 | 47200001 | 47440000 | *KLHL1* |
| 15 | 11 | 18400001 | 18480000 | *KIF18B, FAM187A, EFTUD2, CCDC103, C1QL1* |
| 16 | 19 | 7560001 | 7640000 | *DYNC1LI1* |
| 17 | 22 | 10760001 | 10840000 | *PAPSS2* |
