# Supplementary material for: Comparative whole-genome resequencing to uncover selection signatures linked to litter size in Hu Sheep and five other breeds
Source: BMC Genomics. 2024 May 15;25:480. doi: 10.1186/s12864-024-10396-x (PMC11094944; doi:10.1186/s12864-024-10396-x)
Supplement: Supplementary file 5 — Supplementary Material 5 [file 12864_2024_10396_MOESM5_ESM.docx]

**Supplementary Table 5.** KEGG Pathway Enrichment Analysis for candidate genes in putatively selected region selected by XP-CLR and *F*_ST_ in HS vs. others.

| Term | P-Value | Corrected P-Value | Input |
| --- | --- | --- | --- |
| Sulfur metabolism | 0.00413083 | 0.05510956 | *PAPSS2* |
| Selenocompound metabolism | 0.00787279 | 0.05510956 | *PAPSS2* |
| Vasopressin-regulated water reabsorption | 0.01791298 | 0.08359389 | *DYNC1LI1* |
| Cytosolic DNA-sensing pathway | 0.02786162 | 0.08446314 | *ADAR* |
| Salmonella infection | 0.03298439 | 0.08446314 | *DYNC1LI1* |
| Relaxin signaling pathway | 0.05000505 | 0.08446314 | *RXFP2* |
| Purine metabolism | 0.05215837 | 0.08446314 | *PAPSS2* |
| Measles | 0.06179409 | 0.08446314 | *ADAR* |
| Cellular senescence | 0.06214927 | 0.08446314 | *LIN52* |
| Phagosome | 0.06604826 | 0.08446314 | *DYNC1LI1* |
| Hepatocellular carcinoma | 0.06746246 | 0.08446314 | *ARID2* |
| Influenza A | 0.07239697 | 0.08446314 | *ADAR* |
| Neuroactive ligand-receptor interaction | 0.13179525 | 0.14193335 | *RXFP2* |
| Metabolic pathways | 0.44968037 | 0.44968037 | *PAPSS2* |
