# Supplementary material for: Comparative whole-genome resequencing to uncover selection signatures linked to litter size in Hu Sheep and five other breeds
Source: BMC Genomics. 2024 May 15;25:480. doi: 10.1186/s12864-024-10396-x (PMC11094944; doi:10.1186/s12864-024-10396-x)
Supplement: Supplementary file 6 — Supplementary Material 6 [file 12864_2024_10396_MOESM6_ESM.docx]

**Supplementary Table 6.** *FecB* genotypes of the six sheep breeds.

| Populations | number | | *FecB* genotype frequency | | |  | *FecB* allele frequency | |
| --- | --- | --- | --- | --- | --- | --- | --- | --- |
|  |  |  | BB | B+ | ++ |  | B | + |
| HS | | 274 | 0.799(219) | 0.186(51) | 0.015(4) |  | 0.892 | 0.108 |
| LB | | 53 | 0(0) | 0(0) | 1(53) |  | 0 | 1 |
| WX | | 10 | 0(0) | 0.2(2) | 0.8(8) |  | 0.1 | 0.9 |
| OL | | 9 | 0(0) | 0(0) | 1(9) |  | 0 | 1 |
| BM | | 10 | 0(0) | 0(0) | 1(10) |  | 0 | 1 |
| PD | | 10 | 0(0) | 0(0) | 19(10) |  | 0 | 1 |
