# Supplementary material for: Comparative whole-genome resequencing to uncover selection signatures linked to litter size in Hu Sheep and five other breeds
Source: BMC Genomics. 2024 May 15;25:480. doi: 10.1186/s12864-024-10396-x (PMC11094944; doi:10.1186/s12864-024-10396-x)
Supplement: Supplementary file 7 — Supplementary Material 7 [file 12864_2024_10396_MOESM7_ESM.docx]

**Supplementary Table 7.** Putatively selected region in HR vs. LR groups of Hu sheep.

| Chromosome | Position | | Gene name |
| --- | --- | --- | --- |
|  | Start | End |  |
| 1 | 139800001 | 139950000 | *U6* |
| 1 | 185100001 | 185400000 | *U7* |
| 1 | 280650001 | 280800000 | *CPNE4* |
| 3 | 225001 | 450000 | *ZMYND19, MRPL41, ARRDC1, DPH7, PNPLA7, EHMT1* |
| 3 | 229875001 | 230175000 | *ATP6V1E1, BID* |
| 4 | 40125001 | 40425000 | *SEMA3D* |
| 4 | 49050001 | 49200000 | *PTPN12* |
| 4 | 50100001 | 50325000 | *PMPCB, DNAJC2, PSMC2, SLC26A5, RELN* |
| 4 | 102600001 | 102825000 | *U6, SSMEM1, ZC3HC1, KLHDC10, TMEM209* |
| 4 | 123900001 | 124125000 | *CRYGN, SMARCD3, WDR86, RHEB, NUB1* |
| 5 | 15150001 | 15300000 | *NDUFA7, KANK3, FBN3, CERS4, RPS28* |
| 5 | 46575001 | 46725000 | *U6, U6, AFF4, HSPA4, ZCCHC10* |
| 6 | 22350001 | 22500000 | *GIMD1, TBCK* |
| 6 | 23625001 | 23775000 | *TET2* |
| 6 | 35625001 | 35850000 | *GRID2* |
| 7 | 62250001 | 62475000 | *U6, DTWD1, FAM227B, GALK2* |
| 7 | 62625001 | 62775000 | *FAM227B, GALK2* |
| 7 | 80025001 | 80250000 | *AKAP5, HSPA2, MTHFD1, ZBTB25, ZBTB1, PPP1R36* |
| 7 | 89550001 | 89700000 | *LTBP2* |
| 8 | 54000001 | 54150000 | *RNGTT* |
| 8 | 65325001 | 65475000 | *TCF21TBPL1SLC2A12* |
| 9 | 41850001 | 42150000 | *CHD7, RAB2A* |
| 9 | 70725001 | 70875000 | *CSMD3* |
| 9 | 85500001 | 85650000 | *MIR599, VPS13B* |
| 10 | 34800001 | 34950000 | *WASF3* |
| 11 | 23700001 | 23850000 | *C17orf98, C17orf98, LASP1* |
| 11 | 27450001 | 27675000 | *SPAG9* |
| 11 | 40275001 | 40425000 | *HIC1, MIR212, MIR132, OVCA2, DPH1, SMG6, RTN4RL1* |
| 12 | 65400001 | 65550000 | *TOR1AIP1, CEP350* |
| 13 | 18600001 | 18825000 | *PARD3* |
| 13 | 22350001 | 22650000 | *PLXDC2* |
| 13 | 52650001 | 52800000 | *HAO1* |
| 15 | 58950001 | 59100000 | *THAP12* |
| 15 | 60750001 | 60975000 | *SLC5A12, ANO3, MUC15* |
| 16 | 3075001 | 3225000 | *RANBP17* |
| 16 | 26700001 | 26850000 | *NDUFS4* |
| 16 | 32775001 | 32925000 | *NNT* |
| 17 | 37575001 | 37725000 | *RPS6KC1* |
| 17 | 40275001 | 40425000 | *SPATA5* |
| 17 | 63450001 | 63750000 | *U6, MYL2, HVCN1, PPP1CC, CCDC63* |
| 17 | 71625001 | 71775000 | *RPLP0, BICDL1, RAB35, GCN1, PXN* |
| 18 | 21750001 | 22050000 | *EFL1* |
| 18 | 69150001 | 69450000 | *RD3L, MIR203B, ASPG, KIF26A, TDRD9* |
| 22 | 25575001 | 25875000 | *U6* |
| 23 | 45300001 | 45600000 | *U7* |
