# Supplementary material for: Comparative whole-genome resequencing to uncover selection signatures linked to litter size in Hu Sheep and five other breeds
Source: BMC Genomics. 2024 May 15;25:480. doi: 10.1186/s12864-024-10396-x (PMC11094944; doi:10.1186/s12864-024-10396-x)
Supplement: Supplementary file 8 — Supplementary Material 8 [file 12864_2024_10396_MOESM8_ESM.docx]

**Supplementary Table 8.** KEGG Pathway Enrichment Analysis for candidate genes in HR vs. LR groups of Hu sheep.

| Term | P-Value | Corrected P-Value | Input |
| --- | --- | --- | --- |
| Ribosome | 0.63462151 | 0.63462151 | *RPLP0* |
| cAMP signaling pathway | 0.61106304 | 0.61672103 | *PPP1CC* |
| Alcoholism | 0.57454 | 0.58527907 | *PPP1CC* |
| Kaposi sarcoma-associated herpesvirus infection | 0.57120802 | 0.58527907 | *BID* |
| Rap1 signaling pathway | 0.5678502 | 0.58527907 | *PARD3* |
| Tuberculosis | 0.56276459 | 0.58527907 | *BID* |
| Influenza A | 0.53823704 | 0.56959065 | *BID* |
| Necroptosis | 0.52361458 | 0.55954891 | *BID* |
| Hepatocellular carcinoma | 0.51234767 | 0.55292966 | *SMARCD3* |
| Hepatitis B | 0.51044437 | 0.55292966 | *BID* |
| Phagosome | 0.50469021 | 0.55292966 | *ATP6V1E1* |
| Hepatitis C | 0.50081694 | 0.55292966 | *BID* |
| Cell adhesion molecules (CAMs) | 0.49100206 | 0.55174458 | *PTPRM* |
| Protein processing in endoplasmic reticulum | 0.48901627 | 0.55174458 | *HSPA2* |
| cGMP-PKG signaling pathway | 0.48702281 | 0.55174458 | *PPP1CC* |
| PI3K-Akt signaling pathway | 0.45861959 | 0.53180356 | *RHEB, RELN* |
| Spliceosome | 0.4540598 | 0.53180356 | *HSPA2* |
| Breast cancer | 0.4540598 | 0.53180356 | *NFKB2* |
| Oxytocin signaling pathway | 0.45193134 | 0.53180356 | *PPP1CC* |
| Apoptosis | 0.45193134 | 0.53180356 | *BID* |
| Phospholipase D signaling pathway | 0.4389869 | 0.53180356 | *RHEB* |
| Natural killer cell mediated cytotoxicity | 0.43680025 | 0.53180356 | *BID* |
| Neuroactive ligand-receptor interaction | 0.42461814 | 0.53180356 | *PARD3, GRID2* |
| Yersinia infection | 0.423502 | 0.53180356 | *PXN* |
| Estrogen signaling pathway | 0.41673674 | 0.53180356 | *HSPA2* |
| Apelin signaling pathway | 0.41673674 | 0.53180356 | *MYL2* |
| Vascular smooth muscle contraction | 0.40759395 | 0.53180356 | *PPP1CC* |
| Osteoclast differentiation | 0.40759395 | 0.53180356 | *NFKB2* |
| Autophagy - animal | 0.40528616 | 0.53180356 | *RHEB* |
| Dopaminergic synapse | 0.40296948 | 0.53180356 | *PPP1CC* |
| Toxoplasmosis | 0.39125131 | 0.53180356 | *HSPA2* |
| Platelet activation | 0.39125131 | 0.53180356 | *PPP1CC* |
| Carbon metabolism | 0.39125131 | 0.53180356 | *HAO1* |
| Metabolic pathways | 0.38914911 | 0.53180356 | *NDUFA7, CERS4, MTHFD1, ATP6V1E1, NDUFS4, HAO1, NNT* |
| Oocyte meiosis | 0.37688853 | 0.53180356 | *PPP1CC* |
| Thyroid hormone signaling pathway | 0.37688853 | 0.53180356 | *RHEB* |
| Pathways in cancer | 0.36133504 | 0.53180356 | *BID, NFKB2, SUFU* |
| C-type lectin receptor signaling pathway | 0.35471286 | 0.53180356 | *NFKB2* |
| Insulin resistance | 0.35220116 | 0.53180356 | *PPP1CC* |
| Inflammatory mediator regulation of TRP channels | 0.33949671 | 0.52864488 | *PPP1CC* |
| NF-kappa B signaling pathway | 0.33434612 | 0.52816996 | *NFKB2* |
| MAPK signaling pathway | 0.33257204 | 0.52816996 | *HSPA2, NFKB2* |
| Glycerophospholipid metabolism | 0.32915576 | 0.52816996 | *PNPLA7* |
| Rheumatoid arthritis | 0.32654557 | 0.52816996 | *ATP6V1E1* |
| ECM-receptor interaction | 0.31334299 | 0.52545209 | *RELN* |
| Dilated cardiomyopathy (DCM) | 0.31067188 | 0.52545209 | *MYL2* |
| Fc gamma R-mediated phagocytosis | 0.30799048 | 0.52545209 | *WASF3* |
| Hypertrophic cardiomyopathy (HCM) | 0.30259665 | 0.52545209 | *MYL2* |
| Cardiac muscle contraction | 0.2971612 | 0.52545209 | *MYL2* |
| Platinum drug resistance | 0.29168379 | 0.52545209 | *BID* |
| Peroxisome | 0.28338833 | 0.52354793 | *HAO1* |
| p53 signaling pathway | 0.27780464 | 0.52208114 | *BID* |
| Viral myocarditis | 0.27217788 | 0.5204805 | *BID* |
| Synaptic vesicle cycle | 0.26934824 | 0.5204805 | *ATP6V1E1* |
| Herpes simplex virus 1 infection | 0.26232163 | 0.51987377 | *BID, RHEB, PPP1CC* |
| Bacterial invasion of epithelial cells | 0.26079379 | 0.51987377 | *PXN* |
| Human immunodeficiency virus 1 infection | 0.25279396 | 0.51987377 | *BID, PXN* |
| Human T-cell leukemia virus 1 infection | 0.24850836 | 0.51987377 | *TBPL1, NFKB2* |
| Long-term potentiation | 0.23749397 | 0.50758516 | *PPP1CC* |
| Lysine degradation | 0.23749397 | 0.50758516 | *EHMT1* |
| Amphetamine addiction | 0.23453083 | 0.50758516 | *PPP1CC* |
| Basal cell carcinoma | 0.22857029 | 0.50758516 | *SUFU* |
| Longevity regulating pathway - multiple species | 0.2195432 | 0.50758516 | *HSPA2* |
| VEGF signaling pathway | 0.21041151 | 0.4985838 | *PXN* |
| Proteoglycans in cancer | 0.20450533 | 0.49535734 | *PPP1CC, PXN* |
| Long-term depression | 0.20426502 | 0.49535734 | *GRID2* |
| Amyotrophic lateral sclerosis (ALS) | 0.19807115 | 0.49535734 | *BID* |
| Sphingolipid metabolism | 0.18869072 | 0.48969734 | *CERS4* |
| Hedgehog signaling pathway | 0.17601425 | 0.46794032 | *SUFU* |
| Basal transcription factors | 0.17281461 | 0.46794032 | *TBPL1* |
| Axon guidance | 0.17113976 | 0.46794032 | *PARD3, SEMA3D* |
| Chemokine signaling pathway | 0.16703593 | 0.46794032 | *PARD3, PXN* |
| Proteasome | 0.16314167 | 0.46794032 | *PSMC2* |
| Cellular senescence | 0.14150121 | 0.42843421 | *RHEB, PPP1CC* |
| Measles | 0.14018198 | 0.42843421 | *HSPA2, BID* |
| Apoptosis - multiple species | 0.14013369 | 0.42843421 | *BID* |
| Nicotinate and nicotinamide metabolism | 0.13344569 | 0.42843421 | *NNT* |
| Glyoxylate and dicarboxylate metabolism | 0.1300824 | 0.42843421 | *HAO1* |
| mTOR signaling pathway | 0.12973039 | 0.42843421 | *RHEB, ATP6V1E1* |
| Hippo signaling pathway | 0.1271474 | 0.42843421 | *PARD3, PPP1CC* |
| Parkinson disease | 0.12586063 | 0.42843421 | *NDUFA7, NDUFS4* |
| Adrenergic signaling in cardiomyocytes | 0.12201971 | 0.42843421 | *MYL2, PPP1CC* |
| Retrograde endocannabinoid signaling | 0.1194757 | 0.42843421 | *NDUFA7, NDUFS4* |
| Insulin signaling pathway | 0.11192732 | 0.42843421 | *RHEB, PPP1CC* |
| Collecting duct acid secretion | 0.10617463 | 0.42843421 | *ATP6V1E1* |
| Sphingolipid signaling pathway | 0.08778616 | 0.39869546 | *CERS4, BID* |
| AMPK signaling pathway | 0.08662495 | 0.39869546 | *RAB2A, RHEB* |
| Human cytomegalovirus infection | 0.07833728 | 0.38812559 | *BID, RHEB, PXN* |
| Leukocyte transendothelial migration | 0.07751059 | 0.38812559 | *MYL2, PXN* |
| Epstein-Barr virus infection | 0.07144133 | 0.38812559 | *PSMC2, NFKB2, BID* |
| One carbon pool by folate | 0.07088793 | 0.38812559 | *MTHFD1* |
| Viral carcinogenesis | 0.07076757 | 0.38812559 | *TBPL1, NFKB2, PXN* |
| Choline metabolism in cancer | 0.06030616 | 0.38666889 | *RHEB, WASF3* |
| Antigen processing and presentation | 0.05523952 | 0.37631924 | *HSPA2, HSPA4* |
| Regulation of actin cytoskeleton | 0.05489574 | 0.37631924 | *MYL2, PPP1CC, PXN* |
| Huntington disease | 0.05079751 | 0.37631924 | *NDUFA7, TBPL1, NDUFS4* |
| Ribosome biogenesis in eukaryotes | 0.04937258 | 0.37631924 | *EFL1, SPATA5* |
| Longevity regulating pathway | 0.04937258 | 0.37631924 | *EHMT1, RHEB* |
| Alzheimer disease | 0.03745451 | 0.3711401 | *NDUFA7, BID, NDUFS4* |
| Tight junction | 0.03456027 | 0.3711401 | *PARD3, MYL2, HSPA4* |
| Legionellosis | 0.03007392 | 0.36422863 | *HSPA2, NFKB2* |
| Non-alcoholic fatty liver disease (NAFLD) | 0.02702236 | 0.36422863 | *NDUFA7, BID, NDUFS4* |
| Oxidative phosphorylation | 0.02192522 | 0.34140703 | *NDUFA7, ATP6V1E1, NDUFS4* |
| Thermogenesis | 0.01725602 | 0.31348443 | *SMARCD3, NDUFA7, RHEB, NDUFS4* |
| Focal adhesion | 0.00929464 | 0.20262324 | *PXN, MYL2, RELN, PPP1CC* |
| mRNA surveillance pathway | 0.00727901 | 0.1983531 | *RNGTT, SMG6, PPP1CC* |
| Endocytosis | 0.00425818 | 0.15471394 | *HSPA2, RAB35, PARD3, GBF1, PSD* |
| Human papillomavirus infection | 0.00369667 | 0.15471394 | *TBPL1, PARD3, ATP6V1E1, PXN, RHEB, RELN* |
| Adherens junction | 0.00308548 | 0.15471394 | *PTPRM, PARD3, WASF3* |
