# Supplementary material for: Comparative whole-genome resequencing to uncover selection signatures linked to litter size in Hu Sheep and five other breeds
Source: BMC Genomics. 2024 May 15;25:480. doi: 10.1186/s12864-024-10396-x (PMC11094944; doi:10.1186/s12864-024-10396-x)
Supplement: Supplementary file 9 — Supplementary Material 9 [file 12864_2024_10396_MOESM9_ESM.docx]

**Supplementary Figure S1.** Decay of LD for six sheep breeds, with one line per breed.
