# Supplementary material for: Comparative whole-genome resequencing to uncover selection signatures linked to litter size in Hu Sheep and five other breeds
Source: BMC Genomics. 2024 May 15;25:480. doi: 10.1186/s12864-024-10396-x (PMC11094944; doi:10.1186/s12864-024-10396-x)
Supplement: Supplementary file 10 — Supplementary Material 10 [file 12864_2024_10396_MOESM10_ESM.docx]

**Supplementary Figure S2.** SNP distribution in 27 chromosomes. Each portion of chromosome is 1 Mb.
