# Supplementary figures and images for: Comparative whole-genome resequencing to uncover selection signatures linked to litter size in Hu Sheep and five other breeds
Source: BMC Genomics. 2024 May 15;25:480. doi: 10.1186/s12864-024-10396-x (PMC11094944; doi:10.1186/s12864-024-10396-x)

**Supplementary Figure S3.** Top 30 enriched signaling pathways of candidate genes during Hu sheep.


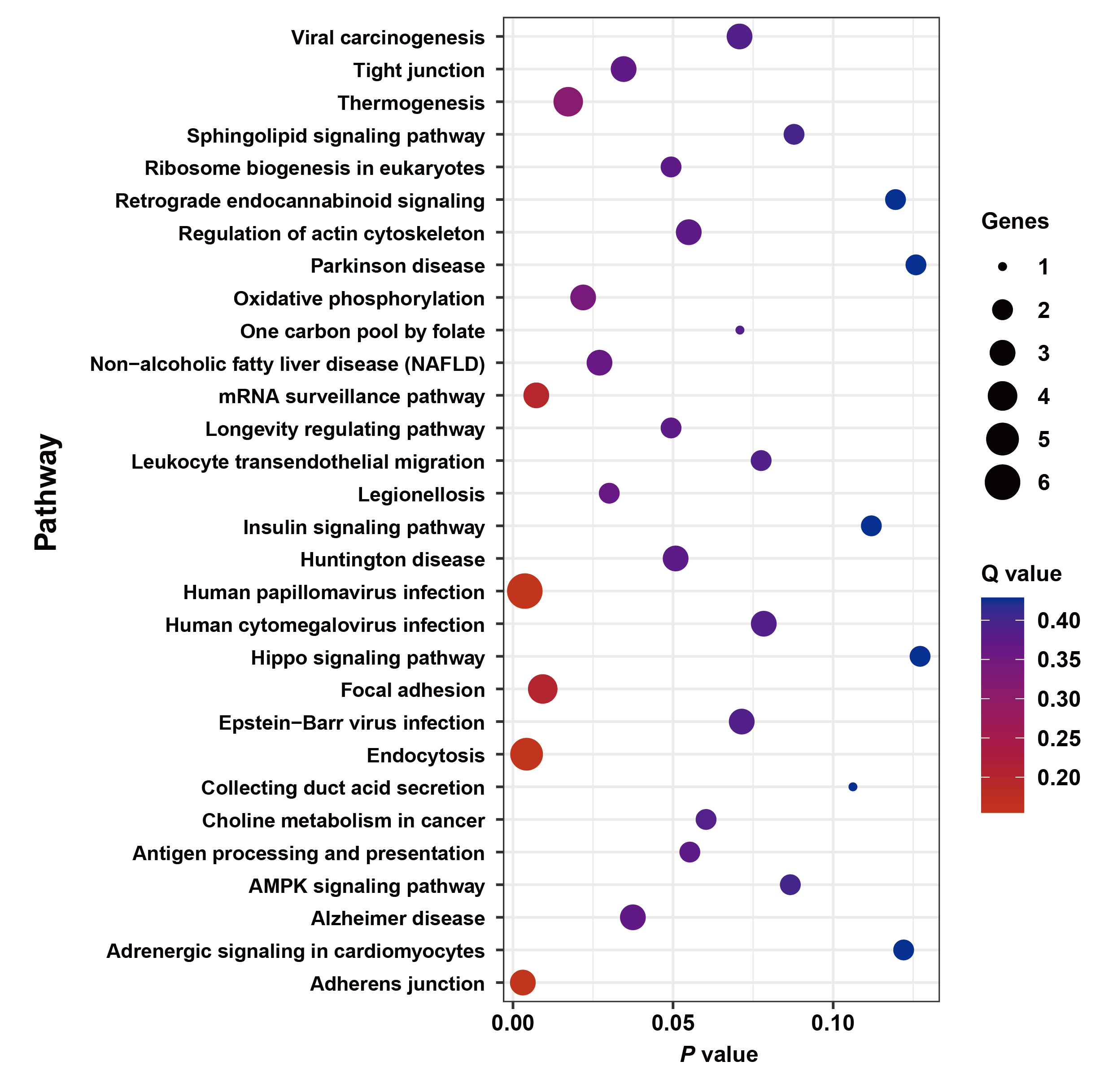

Supplement: Supplementary file 11 — Supplementary Material 11 [file 12864_2024_10396_MOESM11_ESM.docx]
